# Clinical significance of pancreatic calcifications: a 15-year single-center observational study

**DOI:** 10.1186/s40001-022-00725-9

**Published:** 2022-06-25

**Authors:** Wei Wang, Li Chai, Naiyi Zhu, Qingrou Wang, Yiran Zhou, Weimin Chai

**Affiliations:** 1 Department of General Surgery & Research Institute of Pancreatic Diseases, Ruijin Hospital, School of Medicine, Shanghai Jiao Tong University, Shanghai, China; 2 Department of Radiology, Ruijin Hospital, School of Medicine, Shanghai Jiao Tong University, Shanghai, China

**Keywords:** Pancreatic calcifications, Chronic pancreatitis, Non-chronic pancreatitis diseases, Malignancy

## Abstract

**Objectives:**

Pancreatic calcifications (PC) are considered specific for chronic pancreatitis (CP), but PC may also be present in non-CP diseases. The aims are to understand the pattern of calcifications in different diseases and to determine they were related to malignant diseases.

**Methods:**

A retrospective study was performed including patients with PC or CP undergoing surgery in the Department of General Surgery of Ruijin Hospital from January 2003 to June 2018.

**Results:**

PC were observed in 168 (4.5%) of the 3755 patients with pancreatic lesions treated during the study period. The majority of patients with PC had three kinds of CP (73.2%) while 26.8% had other five kinds of non-CP diseases. In patients with non-CP diseases, the incidence of PC in malignant intraductal papillary mucinous neoplasm (IPMN) was significantly higher than benign IPMN (8.3% vs. 0.7*%, p* = 0.004). In patients of CP with pancreatic mass (*n* = 81), PC (Odds ratio = 28.6, *p* = 0.000), advanced age (> 55 years) and parenchymal atrophy were independent predictors for malignancy. In patients of CP without pancreatic mass (n = 110), there were 82 cases (74.5%) with PC and 5 cases (4.5%) with pancreatic ductal adenocarcinoma. The regression model of risk factors was not successful.

**Conclusions:**

The disease spectrum with PC was very diverse. PC may be related to malignant IPMN in non-CP diseases and is related to malignancy in the patients of CP with pancreatic mass and indications for resection.

**Supplementary Information:**

The online version contains supplementary material available at 10.1186/s40001-022-00725-9.

## Introduction

Chronic pancreatitis (CP) is a pathologic fibro-inflammatory syndrome of the pancreas in individuals with genetic, environmental, and/or other risk factors who develop persistent pathologic responses to parenchymal injury or stress [1]. In the course of the disease, patients with CP are at higher risk for the development of pancreatic ductal adenocarcinoma (PDAC) because of the chronic inflammation, with a standardized incidence ratio of 26.3–27, and an overall lifetime risk of 4% [2, 3].

The most widely used diagnostic criteria for CP include the Japanese Pancreas Society (JPS) criteria, Ammann’s (Zurich Workshop) criteria, and Mayo scoring system. Apart from the tissue diagnosis, the presence of stones in the pancreatic ducts and calcifications in the pancreatic parenchyma, have long been considered specific for CP [4, 5].

However, pancreatic calcifications (PC) have also been noted in patients with many other pancreatic diseases [6]. The pattern of calcifications in different diseases is not clear in China, and whether PC were associated with malignancy of pancreatic diseases is not determined because there are very few articles focusing on these issues and most of them have small sample sizes [7, 8].

The present study was conducted to understand the spectrum of diseases that can lead to PC in China, and to explore whether a pattern of PC is associated with malignant pancreatic diseases to provide more targeted information for the selection of other examinations, particularly in the setting of CP and clinical decision-making.

## Materials and methods

### Data source and patient population

Using the electronic and paper records of the Department of General Surgery & Research Institute of Pancreatic Disease in Ruijin Hospital, we performed a retrospective longitudinal cohort study including consecutive patients of CP and PC undergoing surgery from January 2003 to June 2018. All the patients provided written informed consent for the surgical procedures. Every patient underwent detailed evaluation after hospitalization, including laboratory blood tests, serum tumor markers, contrast enhanced CT, contrast enhanced MRI, and EUS or PET/CT if required. A routine multidisciplinary team (MDT) discussion was held for every patient before surgery to determine the individual therapeutic strategy, including the preoperative diagnosis, and indication for resection [9].

### Inclusion and exclusion criteria.

All pancreatic diseases with pancreatic calcification and all chronic pancreatitis (with and without pancreatic calcification) were included. In addition to CP, other pancreatic diseases without pancreatic calcification were excluded.

All the diagnoses of CP or non-CP diseases and their malignancies were confirmed histopathologically. If malignant disease (such as PDAC) coexists with CP, it was called malignant CP. We applied WHO-defined criteria to confirm the diagnosis of malignant SPT, namely, angioinvasion, perineural invasion, or deep invasion into the surrounding tissue, or metastasis [10, 11].

### Definitions

Calcifications were defined as discrete, hyperattenuating foci on non-contrast CT images. The metrics to measure the pattern of calcifications in and around the pancreatic tumors/neoplasms were as follows: (1) Number (single or multiple) and position (head, body and tail) of calcifications. (2) Location of calcifications—Calcifications were considered intraductal (intraductal stones) if they were located within the main pancreatic or branch ducts and were surrounded by hypodense fluid. Calcifications located within the pancreatic tissue and completely surrounded by it, and apparently not connected with the pancreatic ducts, were considered parenchymal. Intralesional calcification was defined as calcification within the lesion, including wall of the cyst, septa, and mural nodules. Calcification around the pancreatic lesions was defined as calcification pushed aside by the lesion and not assessed as intralesional calcification [12]. (3) Distribution of calcifications—If the calcifications were observed in any particular part of the parenchyma of pancreas, such as the uncinate process, head, body, or tail of the pancreas, then it was considered to have segmental distribution while in patients with diffuse distribution the calcifications were located throughout the entire parenchyma of pancreas [7]. (4) Morphology of calcification—Eggshell calcifications was defined as linear calcium deposits in the wall or septae. Punctate or point calcifications were defined as calcifications of < 5 mm in diameter, and coarse calcifications were defined as calcifications of > 5 mm. “Cyst wall” indicates calcification that was found beside the cyst, and “septum” indicates calcification that was present in the cyst bulkhead. A central calcified scar consisted of calcified septations radiating outwards with a sunburst or stellate appearance called as stellate central calcifications [6, 8, 12].

Pancreatic cystic lesions were defined as those exhibiting a round or oval shape with a recognizable wall, and attenuation similar to that of water with no enhancement after the administration of contrast medium, and the presence of enhancing solid components in less than 50% of the lesion. A solid lesion was defined as a discrete mass of soft-tissue attenuation with the enhancing solid portion involving more than 90% of the lesion. A solid and cystic lesion was defined as a lesion in which the enhancing solid component involved 50–90% of the lesion [13].

The main pancreatic duct (MPD) was labelled as dilated if its diameter was more than ≥ 4 mm in the head of pancreas, ≥ 3.5 mm in the body, or ≥ 1.5 mm in tail region [14, 15]. Pancreatic atrophy was deemed present when the anteroposterios width of the pancreas was less than 1 cm in more than half of the pancreas and was confirmed histopathologically [16]. The common duct was considered dilated when its transverse short-axis dimension was > 7 mm [17].

Patients were labelled as smokers if they smoked ≥ 100 cigarettes in their lifetime. Patients were considered alcohol drinkers if they consumed ≥ 15 drinks (100–150-g alcohol) per month on an average and no binges [18].

Suspicious lymph nodes were defined as those with a short axis diameter > 1 cm, abnormal round morphology, heterogeneity, or central necrosis. Vascular invasion was said to be present if the tumor abutment was more than 90° of the circumferences of the superior mesenteric artery, total occlusion of major peripancreatic arteries, or the tumor abutted more than 180° of the circumferences of the portal vein or superior mesenteric vein [19].

### CT examination

The CT images were first screened and evaluated by radiologist (Li Chai). Then the results were submitted to two radiologists for further evaluation, with 5 years (Qingrou Wang) and 13 years (Naiyi Zhu) of experience in this field, respectively. The discrepancies between the three readers’ interpretations were submitted to an imaging seminar, which was led by a senior radiologist (Weimin Chai) with more than 25 years of experience in this field, to resolve the discrepancies by consensus. The radiologists were both blinded to the pathology results and the clinical outcomes [8]. All discussion and results were recorded by the junior radiologist and approved by the team members.

For routine CT examination of the upper abdomen, we used a threshold of > 160 HU to indicate calcification and patients were placed in a supine position, and scanned from the top of the diaphragm to the lower edge of the both kidneys. Patients were kept fasting for more than 8 h and were asked to drink about 800–1000 ml of water before the CT examination to fill the upper gastrointestinal tract. Conventional upper abdominal CT scanning protocol included spiral scanning, 120 kVp, dynamic mA technology, noise index (NI) (about 10–12 Hu), thickness/interval (2.5–5 mm), and the pitch (less than 1). For enhanced CT examination of the upper abdomen, patients were asked to breath-hold, followed by injection of 80–100 ml of non-ionic iodine contrast agent (about 1.5 ml/kg) with high pressure syringe at 2.5–3.5 ml/s flow rate, and images were obtained in arterial phase (35–40 s after the onset of injection) and a venous phase acquisition (60–70 s).

### Statistical analysis

Quantitative data were presented as mean ± standard deviation (mean ± SD), and analyzed by the *t* test if normally distributed, or by the Wilcoxon rank-sum test in case of skewed distribution. Categorical data were presented as frequency (percentage) and analyzed by a *χ*^2^-test, with or without Yates’ correction for continuity or Fisher’s exact test where appropriate. The determination of our variables was based on previous studies, and are shown in Additional file 10: Table S1 and later in Additional file 10: Table S4 [7, 8, 20]. Multiple logistic stepwise regression analysis was used to determine the independent risk factors for the development of PDAC in patients with CP. All the statistical analyses were performed using the SPSS version 22.0 software (Statistical Packages for Social Sciences, Chicago, IL, USA). A *p* value of < 0.05 was considered statistically significant.

## Results

### Patients

**Fig. 1:**
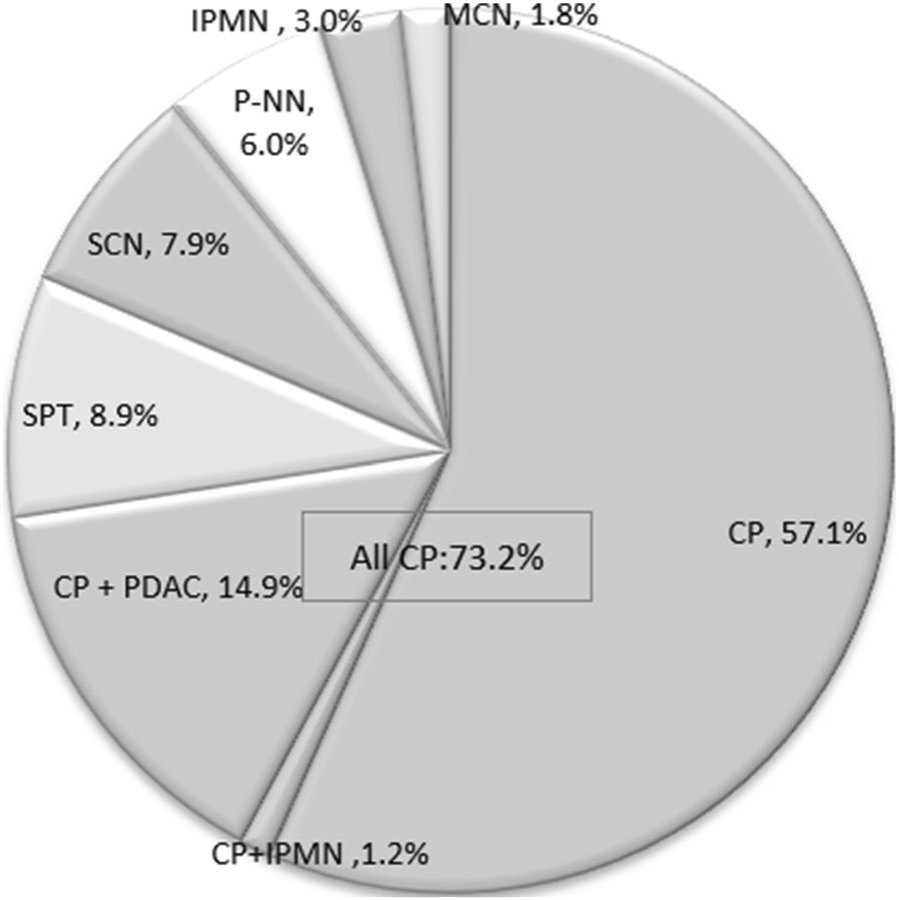
Disease spectrum of patients with pancreatic calcifications. *CP* Chronic PANCREATITIS, *IPMN* intraductal papillary mucinous neoplasm, *PDAC* pancreatic ductal adenocarcinoma, *SPT* solid pseudopapillary tumors, *SCN* serous cystic neoplasm, *P*-*NN* pancreatic neuroendocrine neoplasm, *MCN* mucinous cystic neoplasm

PC were observed in 168 (4.5%) of the 3755 patients with pancreatic lesions treated during the study period and the incidences of PC in the pancreatic diseases were lower than these in previous studies (Table 1). Among these 168 cases with PC, the most common disease was three kinds of CP (73.2%), including CP alone (57.1%), CP combined with intraductal papillary mucinous neoplasm (IPMN) (1.2%) and CP combined with PDAC (14.9%). Other non-CP diseases (26.8%) included five pancreatic diseases, such as pseudopapillary tumors (SPT), serous cystic neoplasm (SCN), Pancreatic neuroendocrine neoplasm (P-NN)**,** IPMN, and mucinous cystic neoplasm (MCN) (Fig. 1).

**Table 1:** Histological diagnosis of patients with pancreatic calcifications (PC)

	*n*	PC		
		*n*	%	Previous study
CP^a^	204^b^	123	60.8	68% [4]
SPT	223	15	6.7	30% [3]
SCN	381	12	3.1	30% [3]
P-NN	345	10	2.9	20–22% [3]
IPMN^c^	363	5	1.4	20–80% [3]
MCN	187	3	1.6	15% [3]
	3755	168	4.5	–

^a^ Including the 34 patients with CP and PDAC (Calcification, *n* = 25; no Calcification, *n* = 9) and the two patients with IPMN combined with calcification

^b^There were 41 cases (43.6%) with PC and 29 cases (30.9%) with PDAC in cases with a pancreatic mass (*n* = 94), and 82 cases (74.5%) with PC and 5 cases (4.5%) with PDAC in cases without pancreatic mass (*n* = 110)

^c^Not included two patients with CP combined IPMN, and 4 out of 5 cases were malignant. *CP* chronic pancreatitis, *IPMN* intraductal papillary mucinous neoplasm, *PDAC* pancreatic ductal adenocarcinoma, *SPT* solid pseudopapillary tumours, *SCN* serous cystic neoplasm, *P*-*NN*: pancreatic neuroendocrine neoplasm, *MCN* mucinous cystic neoplasm

### CT features of PC

In the patients with non-CP diseases, there were no intraductal stones. Segmental PC(s) in the lesion or around the lesion in pancreatic parenchyma was the major feature of these non-CP diseases. Pancreatic intraductal stones combined with parenchymal calcifications was the major feature of CP and malignant IPMN (Additional file 10: Tables S2).

Among the 155 patients with PC whose imaging were performed in our hospital, the sensitivity and specificity of combined pancreatic intraductal and parenchymal calcifications for the diagnosis of CP or CP combined with IPMN was 72.7% (80/110) and 100% (45/45), respectively. (Additional file 10: Table S3).

The presence of diffuse distribution of parenchymal calcifications alone and intraductal stones alone ruled out malignancy with high specificity (95.1% and 94.3%, respectively). (Additional file 10: Table S3). The difference in the incidence of PC was significantly lower in non-malignant IPMN than malignant IPMN (*p* = 0.002). There was no difference between the non-malignant and malignant MCN group (2.5% vs. 3.6%, *p* = 0.560) (Table 3).

### PC and malignancy

Among the malignant diseases, such as malignant CP, P-NN, IPMN, and MCN, the incidence of PC was significantly higher in patients with malignant IPMN compared to non-malignant IPMN (8.3% vs. 0.7*%, p* = 0.002) (Table 3).

### Independent predictors of malignancy in CP

In the series of 204 patients with CP, there were 34 cases with PDAC (malignant CP group). (Table 1, Additional file 10: Table S1) Using a stepwise—logistic regression analysis, only the age at admission (> 55 years), parenchymal atrophy, body mass index (> 24 kg/m^2^), PC and the presence of pancreatic mass were shown to be the independent risk factors for the development of PDAC (Table 4).

The cases with pancreatic mass underwent further analysis. Using univariate analysis, multiple factors were shown to differentiate non-malignant vs. malignant pancreatic mass as shown in Additional file 10: Table S4. On multivariate logistic regression analysis, PC [OR (Odds ratio) = 28.6, *p* = 0.000], advanced age (> 55 years) and parenchymal atrophy were found to be the independent predictors of PDAC. CBD dilation or CBD stent implantation was a protective factor (OR = 0.2*, p* = 0.032) (Table 4).

## Discussion

The results of our cohort study suggest that the majority (73.2%) of patients with PC had CP and 26.8% had other pancreatic diseases. This observation is consistent with that of the previous study by Campisi et al*.* [7] But the disease spectrum was more diverse in our study than that reported by Campisi et al. In the CP spectrum, there were three kinds of CP, including non-malignant CP, malignant CP and CP combined with IPMN in our study. The non-CP diseases include not only the P-NN, the IPMN, the SCN, but also the SPT and the MCN. In addition to that, the rate of malignant CP was high (14.9% in our series vs. 3.9% in Campisi’s study) (Fig. 1, Table 1). On the other hand, except for CP, the results of our cohort study showed that the incidences of PC in the pancreatic diseases were lower than these in previous studies [3, 4] (Table 1), which further showed that there were some differences in the case composition of pancreatic diseases between the East and the West.

One of the key features of CP is the presence of pancreatic intraductal stones and diffuse distributed parenchymal calcifications. Together, these features have high sensitivity (72.7%) and specificity (100%) for diagnosing CP. (Additional file 10: Table S3). All our findings are similar to the revised Japanese clinical diagnostic criteria for CP [4]. However, our series also showed that there is a small possibility of presence of P-NN or malignant IPMN which can mimic CP (Additional file 10: Table S2).

The other key points for the differentiation of CP and non-CP were the presence of typical features of the non-CP diseases. (Additional file 10: Tables S2, Additional file 1: Figure S1, Additional file 2: Figure S2, Additional file 3: Figure S3, Additional file 4: Figure S4, Additional file 5: Figure S5, Additional file 6: Figure S6, Additional file 7: Figure S7, Additional file 8: Figure S8, Additional file 9: Figure S9). The curvilinear or peripheral eggshell-like calcifications were the main feature of SPT. Point or block calcification(s) at the edge of the lesion was found in malignant IPMN (80%), P-NN (60%), MCN (66.7%) and SPT (20.0%). Stellate central calcification was the main finding of SCN (Table 2, Additional file 10: Table S2). These findings of SPT were slightly different from that of the previous studies, where the calcifications of SPT were characteristically peripheral and punctate [21]. The calcifications of P-NN were usually focal, coarse, irregular, and centrally located [22, 23], calcifications of MCN were peripheral eggshell or focal punctate calcifications, [24] calcifications of IPMN were punctate calcifications (87%) and coarse calcification (33%) [25, 26]. In our series, one of the important identification points was that calcifications of pseudocyst are located in the cystic wall, whereas in the non-CP diseases, the cystic pancreatic lesions usually have only segmental calcifications.

**Table 2:** Pancreatic calcifications on computed tomography

Cal. morphology ^a^	Pse. + Cal.^b^ (*n* = 7)	SPT (*n* = 15)	SCN (*n* = 12)	P-NN (*n* = 10)	IPMN ^c^ (*n* = 5)	MCN (*n* = 3)
Eggshell-like	0	12 (80%)	0	0	0	0
Point cal. at the edge	7 (100%)	3 (20%)	0	6 (60%)	4 (80%)	2 (66.7%)
Stellate central cal	0		9 (75%)	0	0	0
Central cal	0		0	2 (20%)	0	0
Septal cal	0		3 (25%)	0	0	1 (33.3%)
Not at the site of lesions^d^	0		0	2 (20%)	1 (20%)	0

^a^The definitions of calcification(s) morphology were show in the first part of “Definitions”; cal., calcification(s)

^b^Pancreatic pseudocyst whose pancreatic calcification(s) at the site of or the edge of the cystic lesions

^c^Malignant IPMN

^d^Point calcification(s) does not at the site or the edge of the solid or cystic lesions

*SPT* solid pseudopapillary tumors; *SCN* serous cystic neoplasm; *P*-*NN* pancreatic neuroendocrine neoplasm; *IPMN* intraductal papillary mucinous neoplasm; *MCN* mucinous cystic neoplasm

The second important purpose of this study was to determine whether PC can be used to suggest a diagnosis of malignancy. On a whole, the presence of diffuse distribution of parenchymal calcifications alone and intraductal stones alone ruled out malignant with high specificity (95.1% and 94.3%, respectively) (Additional file 10: Table S3). However, in our study group, the majority of the patients had CP and IPMN with only one case of mixed ductal–endocrine carcinoma of the pancreas and mucinous cystadenocarcinoma each. In addition, the difference in the incidence of PC was significantly lower in non-malignant IPMN than malignant IPMN (*p* = 0.002) in our study. Previous studies have also reported that the presence of coarse calcifications, when combined with other morphological features, such as main ductal dilatation, solid nodules, or size > 3 cm, might be a radiological sign of malignancy [27]. Furthermore, a study by Tsujimae et al. found that pancreatic calcification was significantly associated with invasive intraductal papillary mucinous carcinoma, showing that pancreatic calcification might be a predictor of invasive malignant IPMN [12]. In our series, five (8.3%) cases of malignant IPMN had many kinds of calcifications (Tables 1, 2, 3, Additional file 10: Table S2, Additional file 2: Figure S2, Additional file 3: Figure S3, Additional file 4: Figure S4), showing that calcification is only a warning factor for malignancy and is not a reliable indicator for malignant.

**Table 3:** Pancreatic calcifications and malignancy of diseases^a^

	Non-malignant		Malignant		*P* value
		Calcification		Calcification	
CP (n = 191)	157	85 (54.1%)	34	25 (73.5%)	0.038
P-NN (*n* = 345)	334^b^	9 (2.7) ^b^	11 ^c^	1 (9.1%)^c^	0.280
SPT (*n* = 223)	177	13 (7.3)	31	0 (0.0%)	0.224
IPMN (*n* = 363) ^d^	303	2 (0.7%)	60	5 (8.3%)	0.002
MCN (*n* = 187)	159	4 (2.5%)	28	1 (3.6%)	0.560

^a^Not included the patients whose preoperative CT examinations were performed at other hospitals.

^b^Pancreatic neuroendocrine tumors (P-NETs)

^c^Pancreatic neuroendocrine carcinoma (P-NEC)

^d^Including the 2 patients with CP and IPMN

*CP* chronic pancreatitis, *P*-*NN* pancreatic neuroendocrine neoplasm, *IPMN* intraductal papillary mucinous neoplasm, *MCN* mucinous cystic neoplasm

The reasons for the occurrence of pancreatic calcification in IPMN are unclear. First, calcification in IPMN is likely to represent a unique and unrecognized form of calcifying obstructive pancreatitis caused by prolonged ductal obstruction by thick mucin, which has a propensity to build up calcium salt deposits and coexist with chronic calcifying pancreatitis [27, 28]. Second, it may also be the result of tumor calcification, as seen in an array of other slow-growing pancreatic neoplasms, such as SCN, MCN, or SPT [28, 29]. Third, tissue hypoxia due to insufficient blood supply can raise cellular pH, diminish the solubility of calcium salts, and result in subsequent precipitation [27]. Finally, pancreatic calcification is associated with a high degree of inflammation and atrophy in the background pancreatic parenchyma, suggesting that pancreatic calcification in patients with IPMN may be caused by chronic pancreatic inflammation. Furthermore, a high degree of inflammation and atrophy in the background pancreatic parenchyma are related to malignant IPMN [12].

Not much is known about PC and malignancy in other non-CP diseases, and some of our results were somewhat contradictory. In MCN, multilocular macrocystic lesions and lesions with calcifications in the wall had a higher risk of malignancy [30]. But in our study we could not find any difference between the non-malignant and malignant group (2.5% vs. 3.6%, *p* = 0.560), which similar to that reported by Wu et al*.* [31]. This may be related to the small number of cases in this study. In a previous study, 7 of 10 P-NN with calcifications had malignant features, unlike the observations of this study [6]. However, in SPT, PC was not useful to differentiate the non-malignant and malignant forms of SPT as found in this study and previous studies (Table 3) [6, 10, 11].

There are few studies showing that PC is associated with cancer [2, 3]. In a study (*n* = 48) reported by Mohamed et al*.*, [8] the presence of a pancreatic mass in CP is suggestive of malignancy, especially when PC are observed. Our results in agreement with these that PC, in combination with other variables, such as advanced age (> 55 years) and atrophy of the parenchyma, was found to a risk factor with the highest odds ratio (28.6, *P* = 0.000) in cases with pancreatic mass. We also found that presence of PC (OR = 4.9), in combination with other variables, such as advanced age (> 55 years), high BMI (> 24 kg/m^2^), atrophy of the parenchyma and pancreatic mass, were seemed to be independent predictors of PDAC for all patients with CP, even the patients had no pancreatic mass. However, because there were many missing values and only five cases of PDAC, the regression model of risk factors was not successful in the group of patients without a pancreatic mass (*n* = 110). Furthermore, the proportion (4.5%, 5/110) of patients with PDAC without a pancreatic mass was similar to the results of previous studies (5–7.1%) [32, 33]. This may be due to the false image caused by the CP itself and the high proportion of cancer in the group of cases with pancreatic masses (29/94). We cannot say that PC is one of the warning signs of CP without pancreatic mass for malignancy in patients who had surgical indications (Table 4).

**Table 4:** Logistic regression analysis for identifying independent risk factors for the development of PDAC in CP

Risk factors	Odds ratio	*P value*
All patients (*n* = 204^a^)		
Age at admission: > 55 years	6.3	0.011
Atrophy of the parenchyma	9.5	0.001
Body mass index: > 24 kg/m ^2^	4.2	0.043
Pancreatic calcification	4.9	0.009
Pancreatic mass	28.8	0.000
Patients with Pancreatic mass (*n* = 81^b^)		
Age at admission: > 55 years	16.9	0.008
Common bile duct (CBD)dilation or CBD stent implantation	0.2	0.032
Pancreatic calcification	28.6	0.000
Atrophy of the parenchyma	8.3	0.007
Patients without Pancreatic mass (*n* = 110^c^)		

*PDAC* pancreatic ductal adenocarcinoma, *CP* chronic

^a^Included the 13 patients whose imaging were performed at other hospitals

^b^Including the data of 25 patients with PDAC, the data of 13 patients were excluded, including those with pancreatic pseudocyst (*n* = 5), these with IPMN (*n* = 2), and these whose imaging examinations were performed at other hospitals (*n* = 6)

^c^Missing values and only five patients with PDAC, which are not enough for establishing a regression model

Common bile duct dilation or bile duct stenting was a protective factor (OR = 0.2*, p* = 0.032). This was contrary to the observations made by previous study [8]. But a similar study by Ruan et al*.* [34] showed that dilated bile ducts passed through the lesions in 79.17% of mass-forming CP and in 16.67% cases of pancreatic carcinoma (*p* < 0.001). The double duct sign, which indicates the expansion of both the pancreatic duct and the bile duct, was similar in both groups. However, the number of cases were small (mass-forming CP, *n* = 24; pancreatic carcinoma, *n* = 30). The rate of calcification in mass-forming CP was significantly more than in pancreatic carcinoma cases (58.3% vs. 10*%, p* < 0.001). In our study, the incidence of calcification in CP with PDAC was more than CP without PDAC cases (75.0% vs. 22.6*%, p* = 0.000 (Additional file 10: Table S4).

About 5–10% of “mass-forming” pancreatitis often masquerades as PDAC. Due to overlap of the imaging findings [35, 36]. In this study, we found that suspicious lymph nodes, abutment or encasement of the arteries around the pancreas, venous involvement, and the tumor size have no ability to differentiate between CP and CP with PDAC. Many other clinical features, such as the duration of symptoms before admission, jaundice, high-colored urine, diabetes mellitus, total bilirubin levels, and the carbohydrate antigen 19–9 (CA19–9), the carbohydrate antigen 125 (CA125) were insignificant using logistic regression, which are in agreement with these in previous studies [20, 37–40]. However, the appearance of these clinical features should raise the suspicion of PDAC and may complement other clinical findings to improve diagnostic accuracy [38].

Our study has some limitations. First, the sample size was relatively small. Second, only the patients undergoing surgery were included. This method guaranteed the accuracy of the diagnosis, but many patients with PC were not included in our study who received medical or endoscopic intervention therapy. Third, because the focus of this study was PC, findings of imaging modalities other than CT were not studied. In addition, the morphological features of bile ducts and pancreatic ducts, including the double duct sign and whether they passed through the lesion or interrupted the lesion areas, were not further evaluated. Fourth, this study was retrospective in nature. The technical parameters adopted in different periods were slightly different. The scan thickness of 5 mm in the early phase of the study could have increase the risk of overlooking PC. Future larger prospective studies are required to validate the findings of this study. Fifth, we were not sure whether the CT scan parameters of other hospitals were consistent with those of our hospital. However, during the pre-operative discussion at our pancreatic center, the imaging data of other hospitals was reviewed and no inconsistency with regards to the presence of PC was found. Sixth, the specific amount of alcohol consumed in previous medical history was not well documented in our series, and there was a lack of detailed information on etiology, such as alcohol intake. We only determined whether the patients were alcoholic drinkers. However, the percentage (20.6%) of alcohol drinkers in all CP cases in our series (Additional file 10: Table S1) was similar to the percentage (18.8%, 405/2153) of patients with alcoholic chronic pancreatitis (alcohol intake: > 80 g/day for men and 60 g/day for women for at least 2 years in the absence of other causes) in a previous study from China, [41] showing that the percentage (20.6%) of drinkers in all CP cases in our series can be regarded as an approximate value of the percentage of patients with alcoholic CP. In addition, among the independent risk factors for the development of PDAC in CP, the risk factors of alcohol drinkers were not included, similar to the results of previous studies from China and Western countries [2, 42]. Finally, all the CP patients, with indications for surgery, had not undergone EUS–FNA/B. In recent years, the application of EUS–FNA/B in the differentiation of mass pancreatitis from PDAC has gradually increased, and relevant data are being investigated.

In summary, the disease spectrum of PC in China, including three kind of CP and five kind of non-CP diseases, was more diverse than these in previous studies. On the other hand, PC may be related to malignant IPMN with indication for resection. In addition, PC, in combination with other variables, were shown to be related to PDAC in the setting of CP with indication for resection, not only in cases with pancreatic mass, but also in cases without pancreatic mass.

## Supplementary Information

**Additional file 1. Supplementary Figure 1. **PC in a patient with SCN.

**Additional file 2. Supplementary Figure 2. **PC in a patient with MCN.

**Additional file 3. Supplementary Figure 3. **PC in a patient with malignant MCN.

**Additional file 4. Supplementary Figure 4. **PC in a patient with SPT.

**Additional file 5. Supplementary Figure 5. **PC in a patient with P-NETs.

**Additional file 6. Supplementary Figure 6. **PC in a patient with P-NEC.

**Additional file 7. Supplementary Figure 7.** PC in a patient with malignant IPMN.

**Additional file 8. Supplementary Figure 8. **PC in a patient with malignant IPMN.

**Additional file 9. Supplementary Figure 9. **PC in a patient with CP combined with PDAC.

**Additional file 10.** Supplementary Tables.

## Data Availability

The data sets to support the findings of this study are included within the article, including Fig. 1, tables and supplementary tables. Any other data used to support the findings of this study are available from the corresponding author upon request.
