# Supplementary figures and images for: Clinical significance of pancreatic calcifications: a 15-year single-center observational study
Source: Eur J Med Res. 2022 Jun 25;27:99. doi: 10.1186/s40001-022-00725-9 (PMC9233388; doi:10.1186/s40001-022-00725-9)

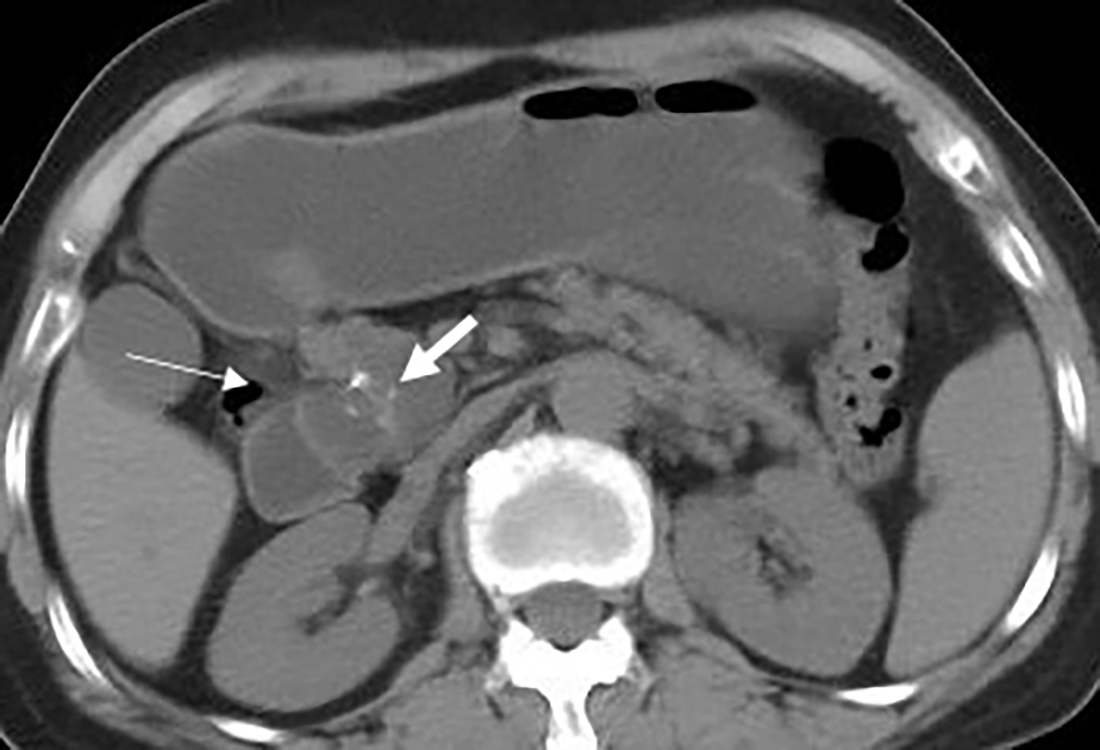

Supplement: Supplementary file 1 — Additional file 1. Supplementary Figure 1. PC in a patient with SCN. [file 40001_2022_725_MOESM1_ESM.zip › Supplementary figure 1-a.jpg]

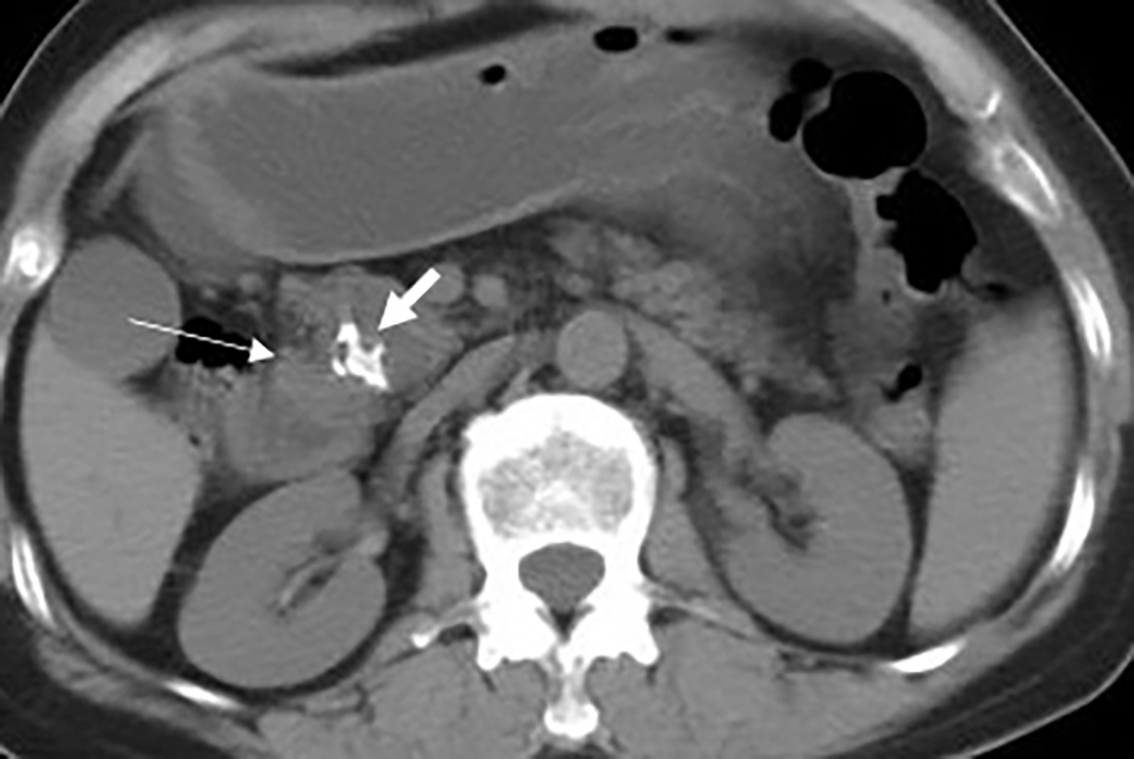

Supplement: Supplementary file 1 — Additional file 1. Supplementary Figure 1. PC in a patient with SCN. [file 40001_2022_725_MOESM1_ESM.zip › Supplementary figure 1-b.jpg]

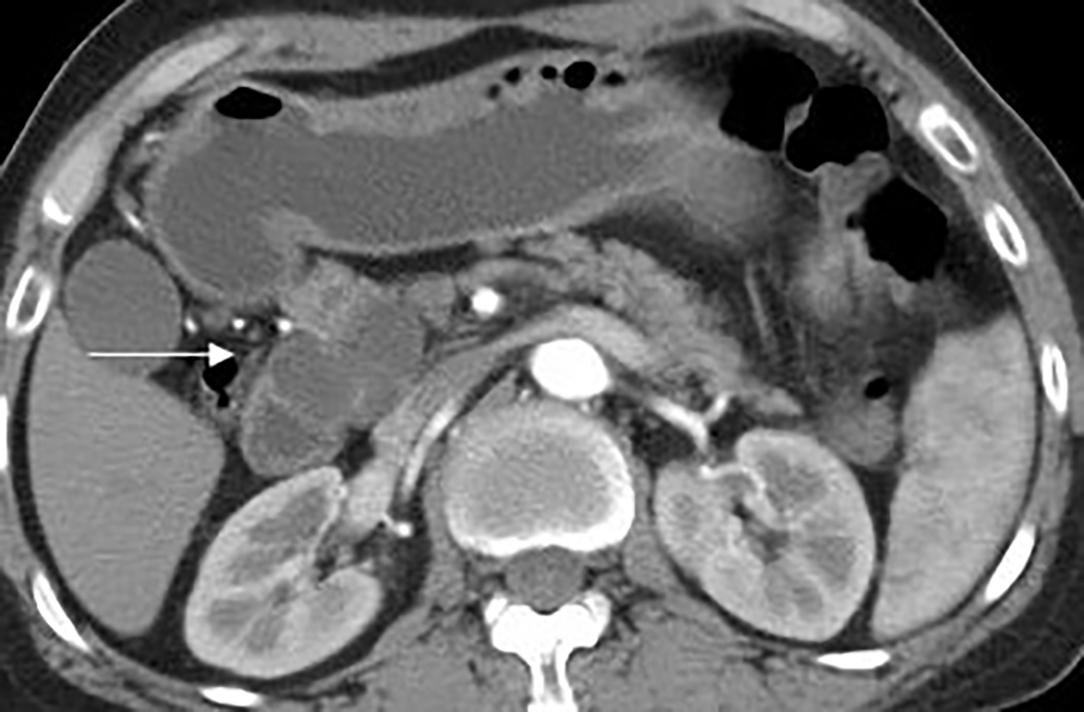

Supplement: Supplementary file 1 — Additional file 1. Supplementary Figure 1. PC in a patient with SCN. [file 40001_2022_725_MOESM1_ESM.zip › Supplementary figure 1-c.jpg]

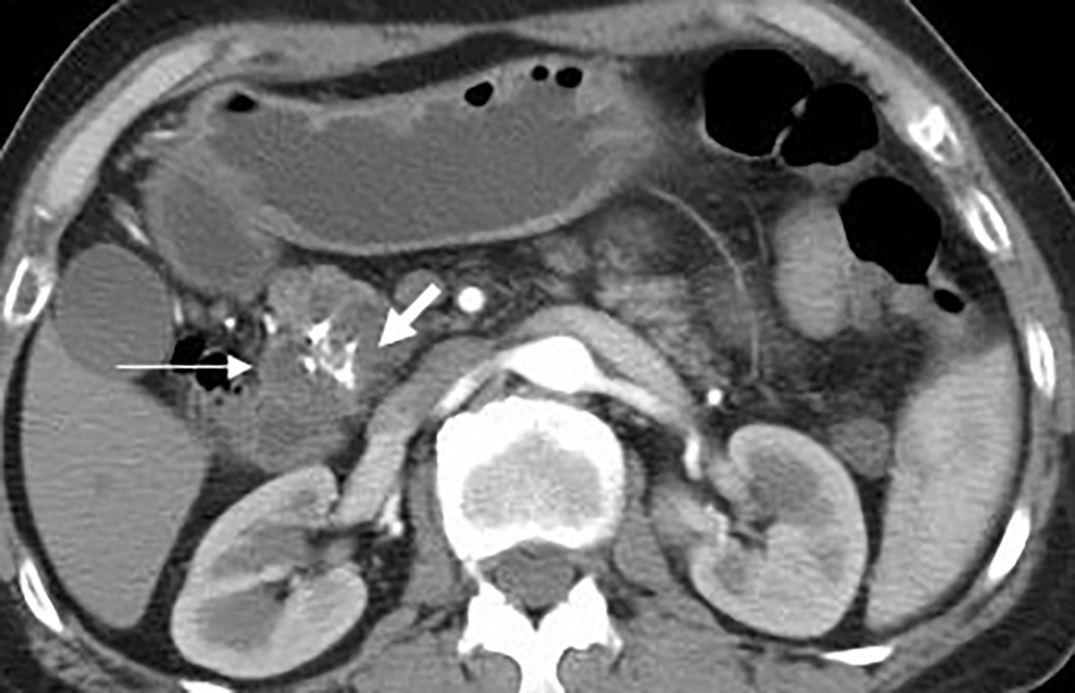

Supplement: Supplementary file 1 — Additional file 1. Supplementary Figure 1. PC in a patient with SCN. [file 40001_2022_725_MOESM1_ESM.zip › Supplementary figure 1-d.jpg]

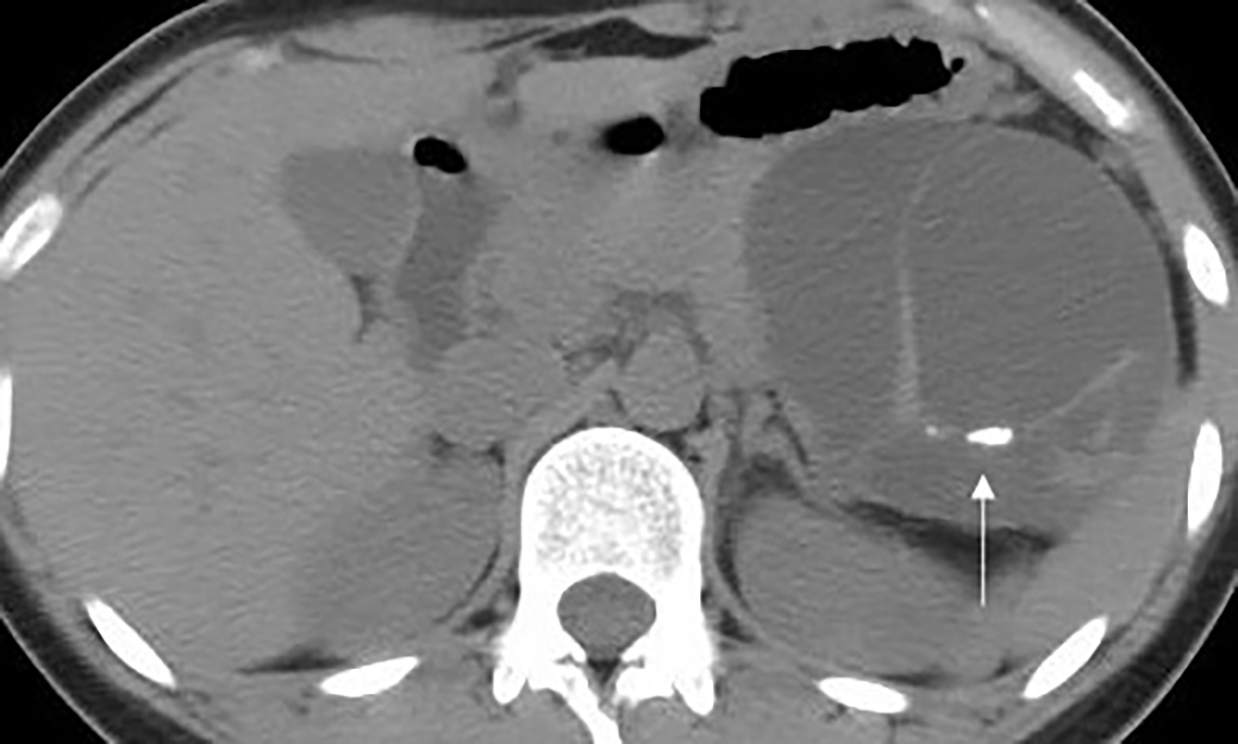

Supplement: Supplementary file 2 — Additional file 2. Supplementary Figure 2. PC in a patient with MCN. [file 40001_2022_725_MOESM2_ESM.jpg]

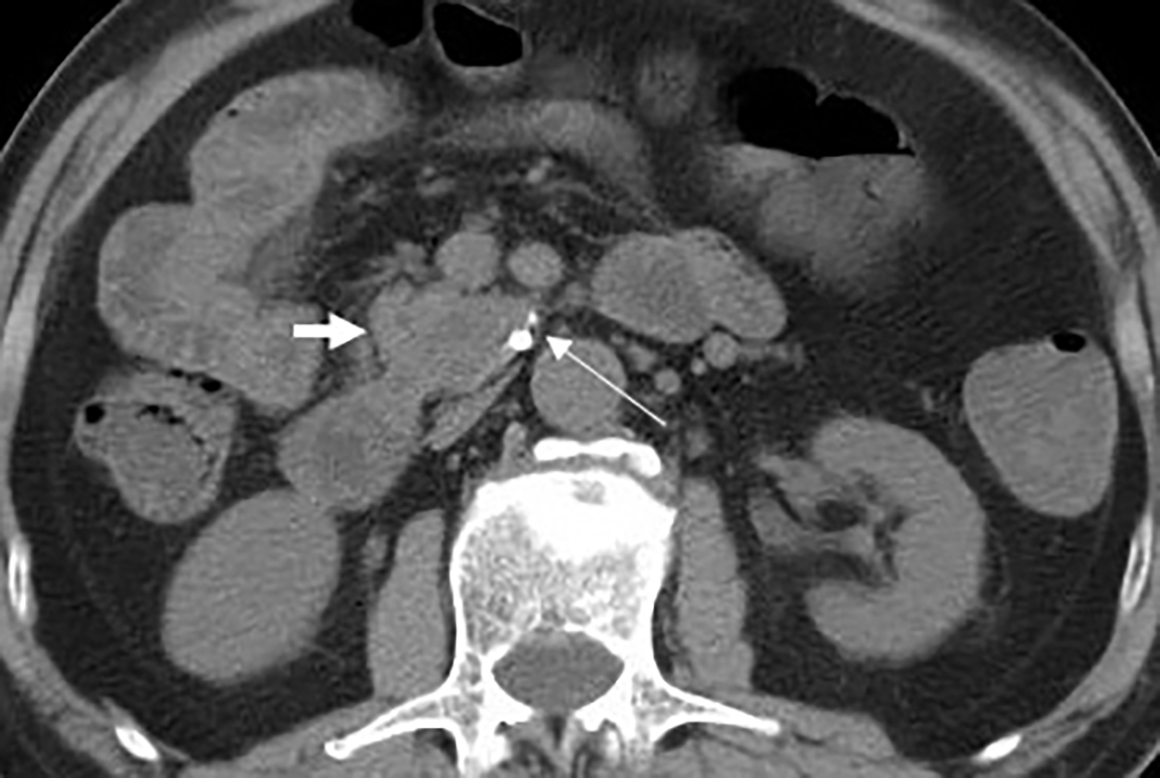

Supplement: Supplementary file 3 — Additional file 3. Supplementary Figure 3. PC in a patient with malignant MCN. [file 40001_2022_725_MOESM3_ESM.zip › 1-Supplementary figure 3-a.jpg]

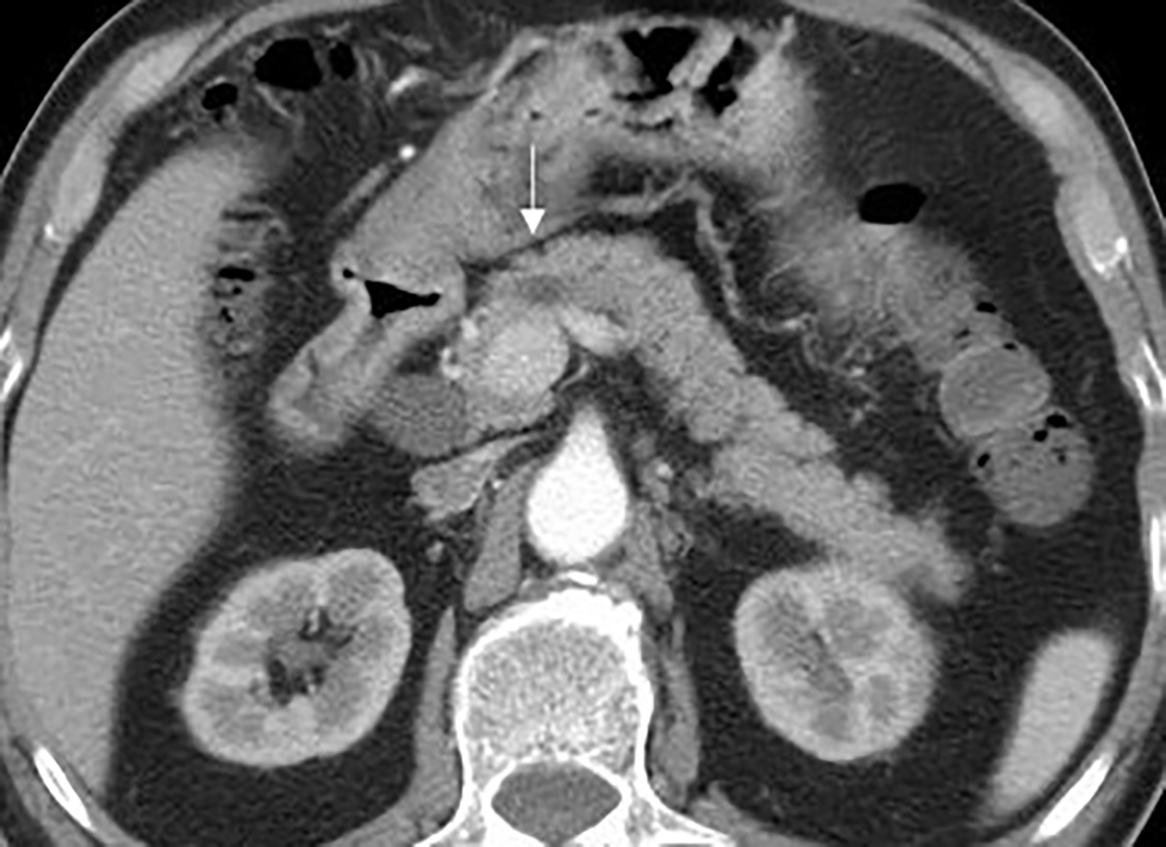

Supplement: Supplementary file 3 — Additional file 3. Supplementary Figure 3. PC in a patient with malignant MCN. [file 40001_2022_725_MOESM3_ESM.zip › Supplementary figure 3-b.jpg]

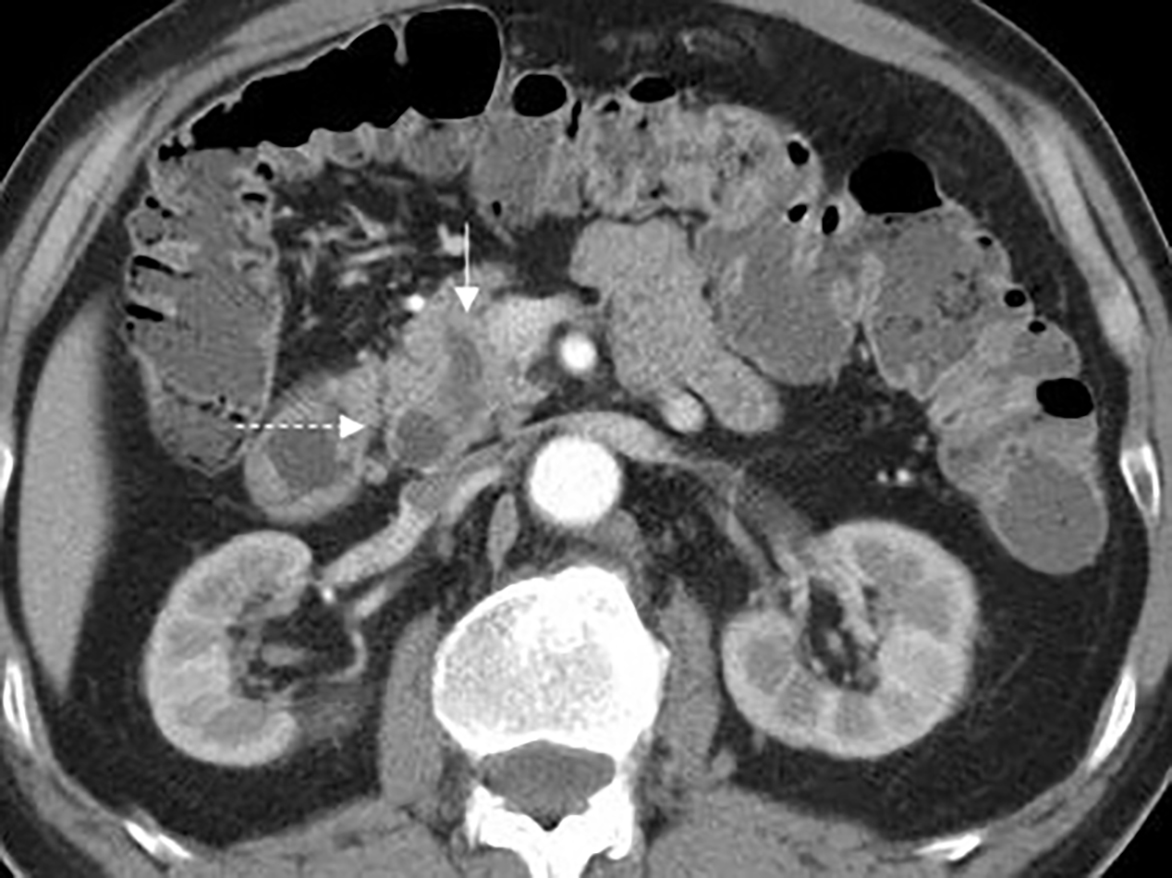

Supplement: Supplementary file 3 — Additional file 3. Supplementary Figure 3. PC in a patient with malignant MCN. [file 40001_2022_725_MOESM3_ESM.zip › Supplementary figure 3-c.jpg]

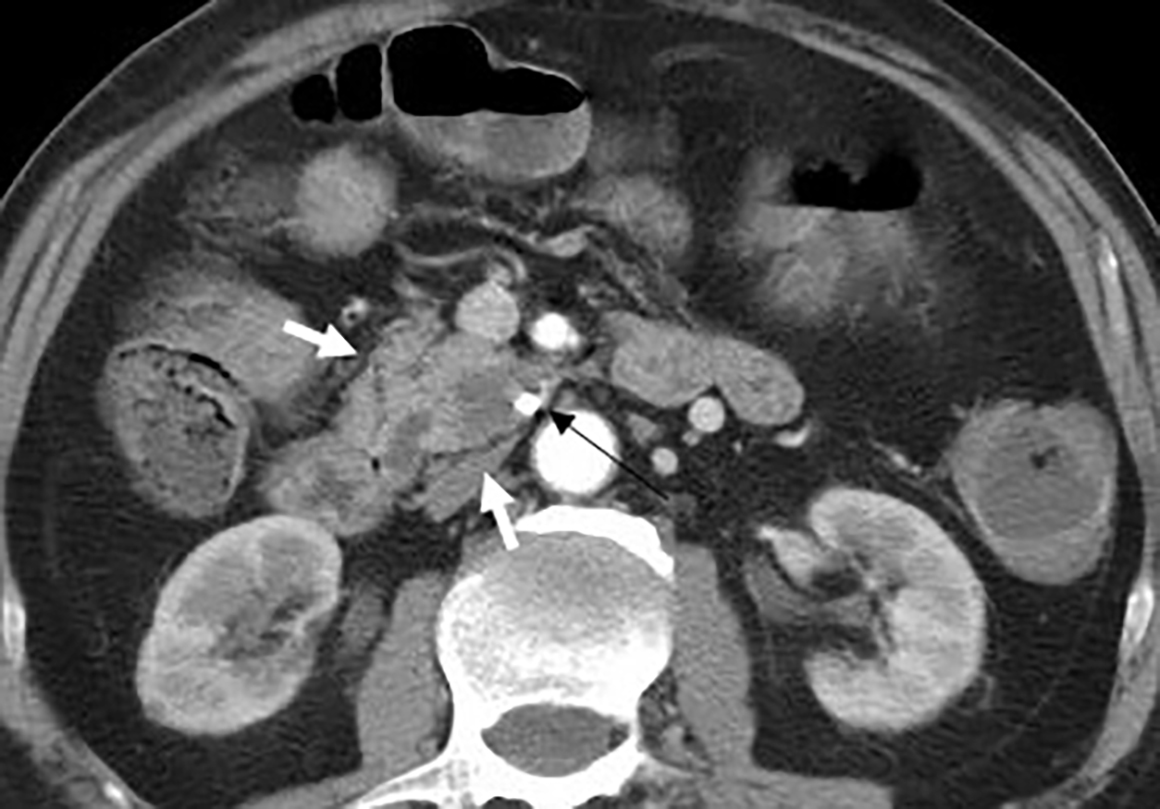

Supplement: Supplementary file 3 — Additional file 3. Supplementary Figure 3. PC in a patient with malignant MCN. [file 40001_2022_725_MOESM3_ESM.zip › Supplementary figure 3-d.jpg]

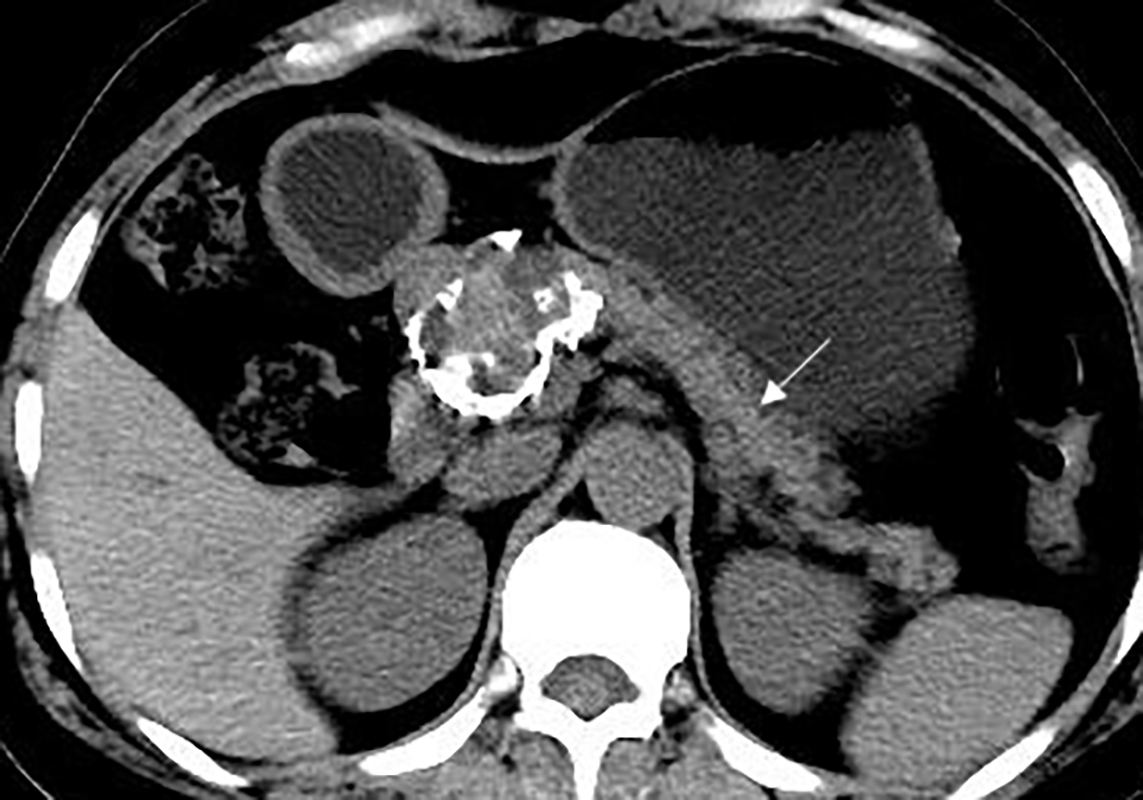

Supplement: Supplementary file 4 — Additional file 4. Supplementary Figure 4. PC in a patient with SPT. [file 40001_2022_725_MOESM4_ESM.zip › Supplementary figure 4-a.jpg]

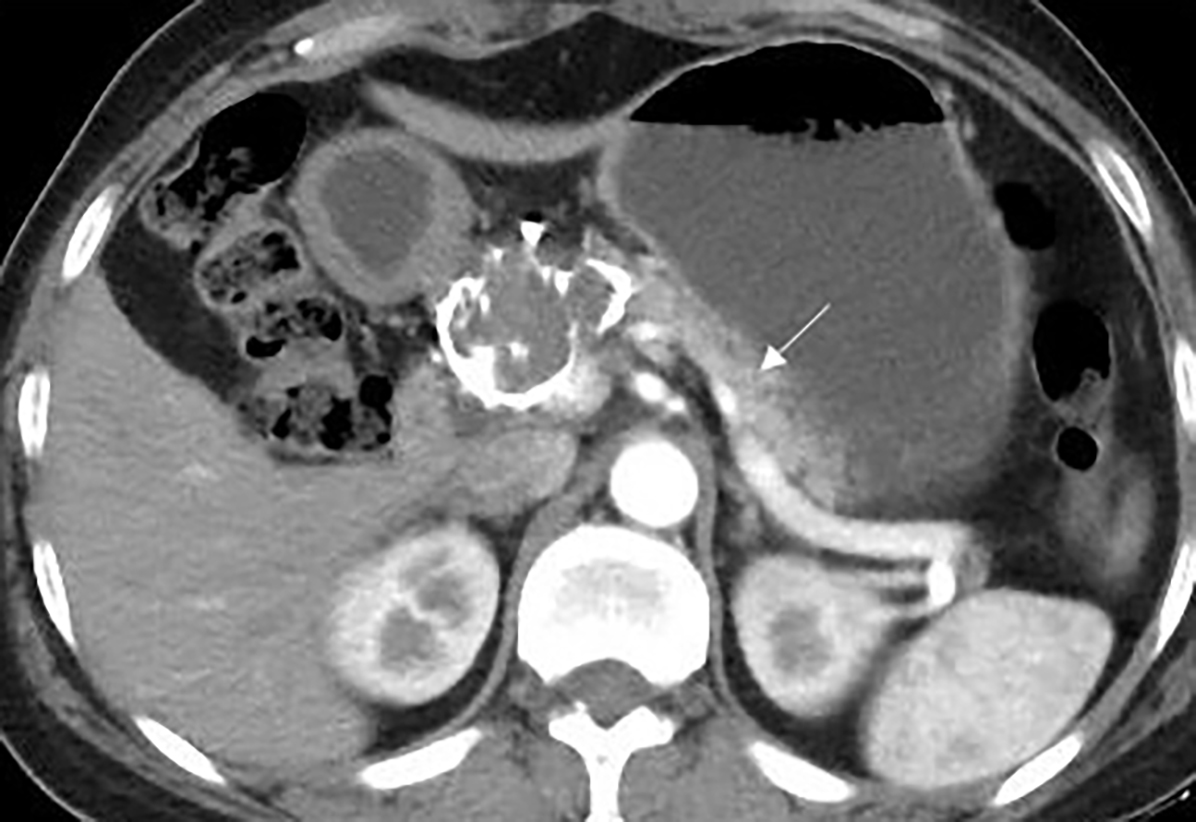

Supplement: Supplementary file 4 — Additional file 4. Supplementary Figure 4. PC in a patient with SPT. [file 40001_2022_725_MOESM4_ESM.zip › Supplementary figure 4-b.jpg]

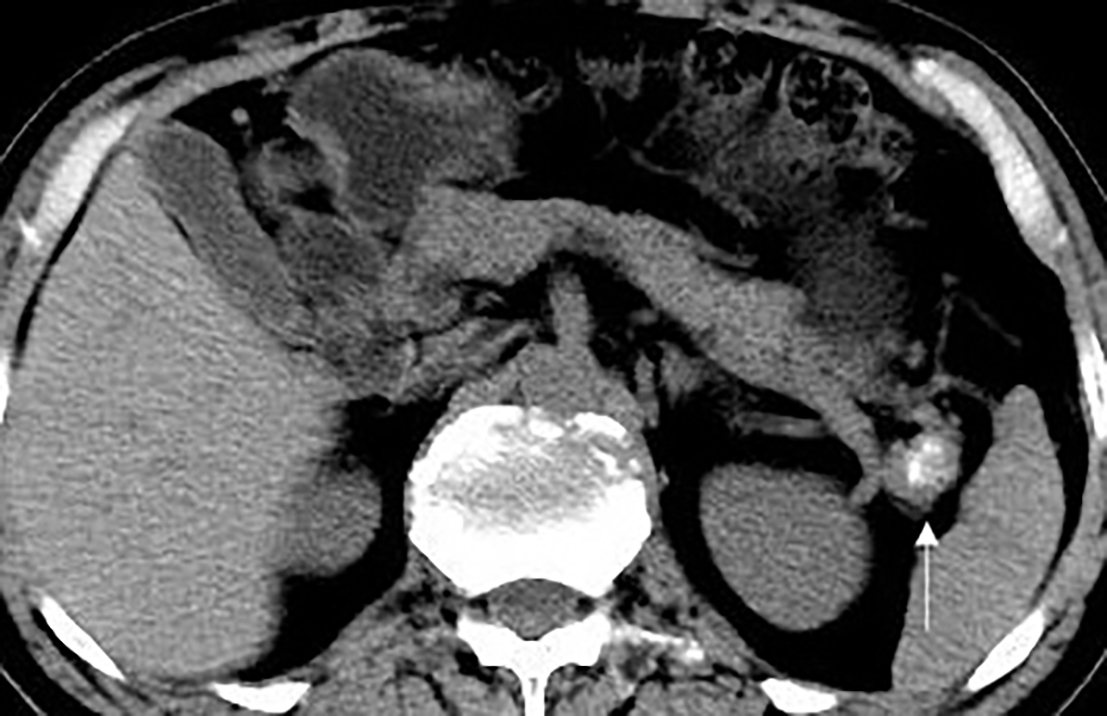

Supplement: Supplementary file 5 — Additional file 5. Supplementary Figure 5. PC in a patient with P-NETs. [file 40001_2022_725_MOESM5_ESM.zip › Supplementary figure 5-a.jpg]

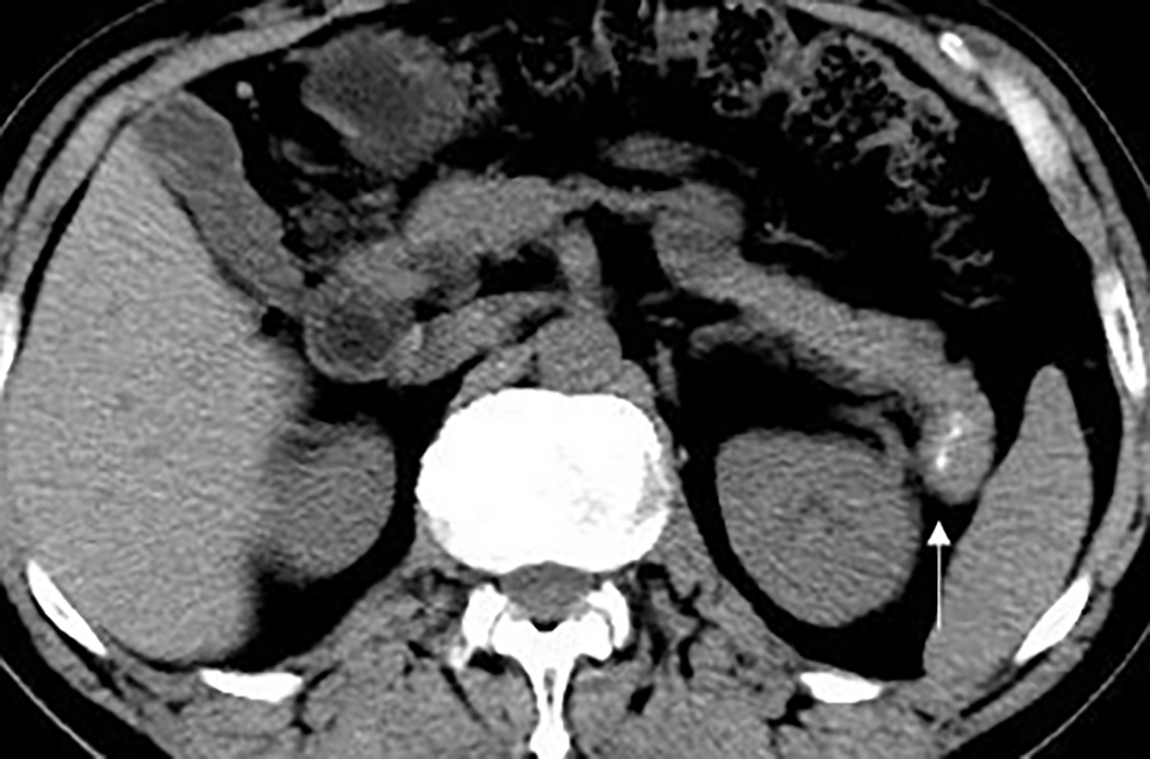

Supplement: Supplementary file 5 — Additional file 5. Supplementary Figure 5. PC in a patient with P-NETs. [file 40001_2022_725_MOESM5_ESM.zip › Supplementary figure 5-b.jpg]

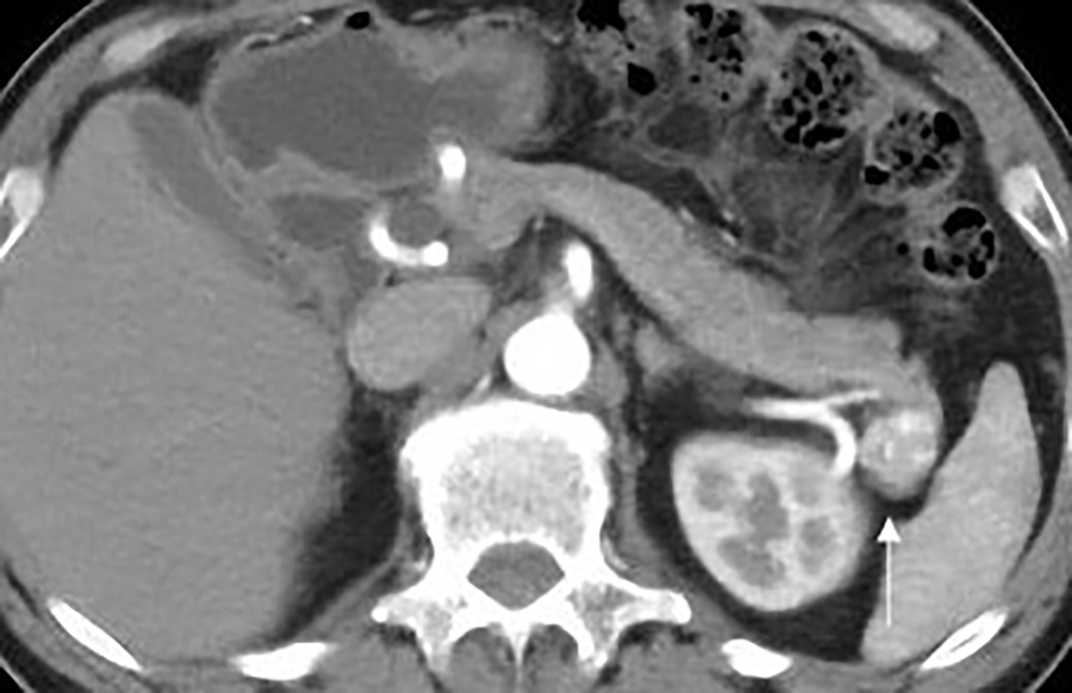

Supplement: Supplementary file 5 — Additional file 5. Supplementary Figure 5. PC in a patient with P-NETs. [file 40001_2022_725_MOESM5_ESM.zip › Supplementary figure 5-c.jpg]

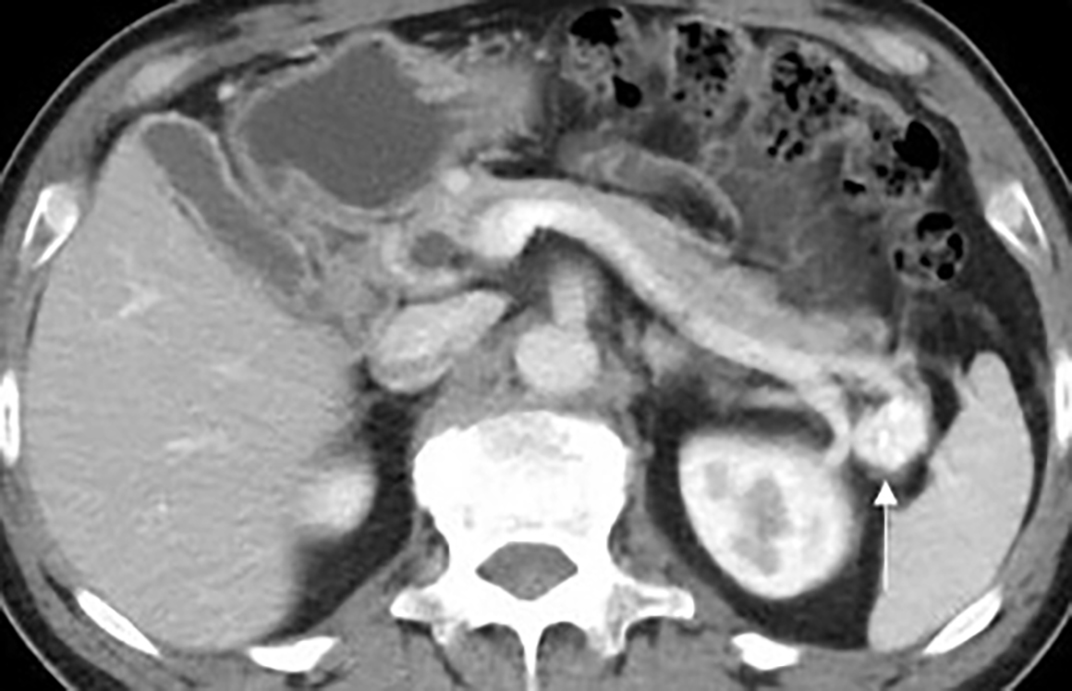

Supplement: Supplementary file 5 — Additional file 5. Supplementary Figure 5. PC in a patient with P-NETs. [file 40001_2022_725_MOESM5_ESM.zip › Supplementary figure 5-d.jpg]

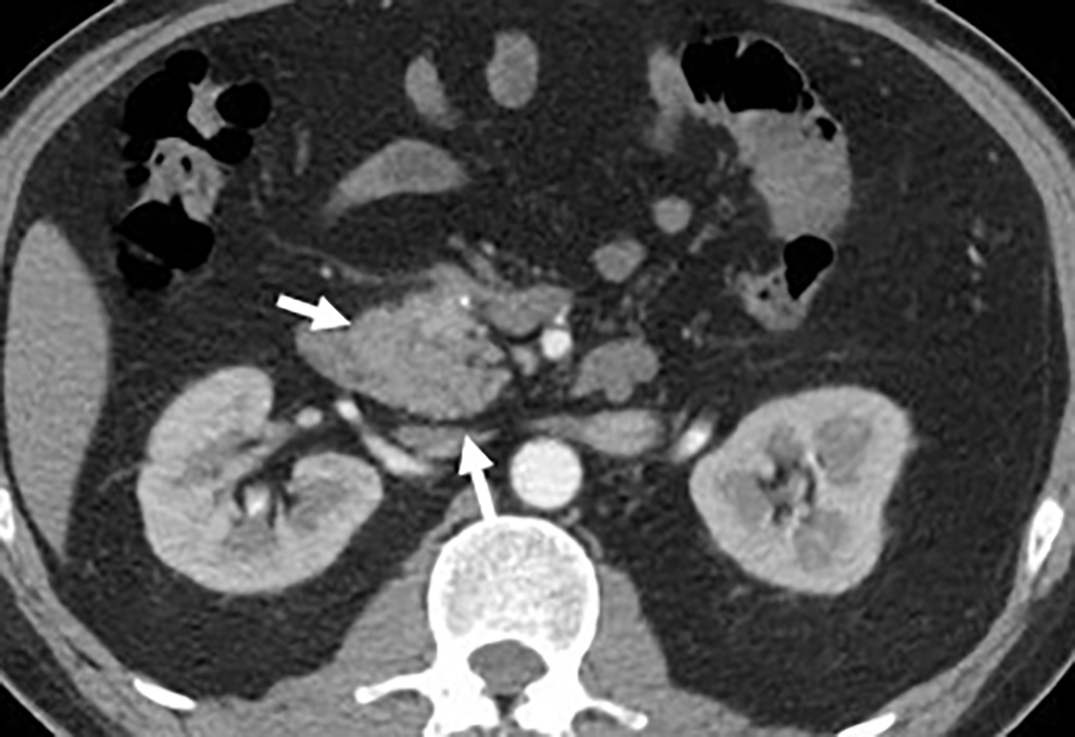

Supplement: Supplementary file 6 — Additional file 6. Supplementary Figure 6. PC in a patient with P-NEC. [file 40001_2022_725_MOESM6_ESM.zip › 2-Supplementary figure 6-d.jpg]

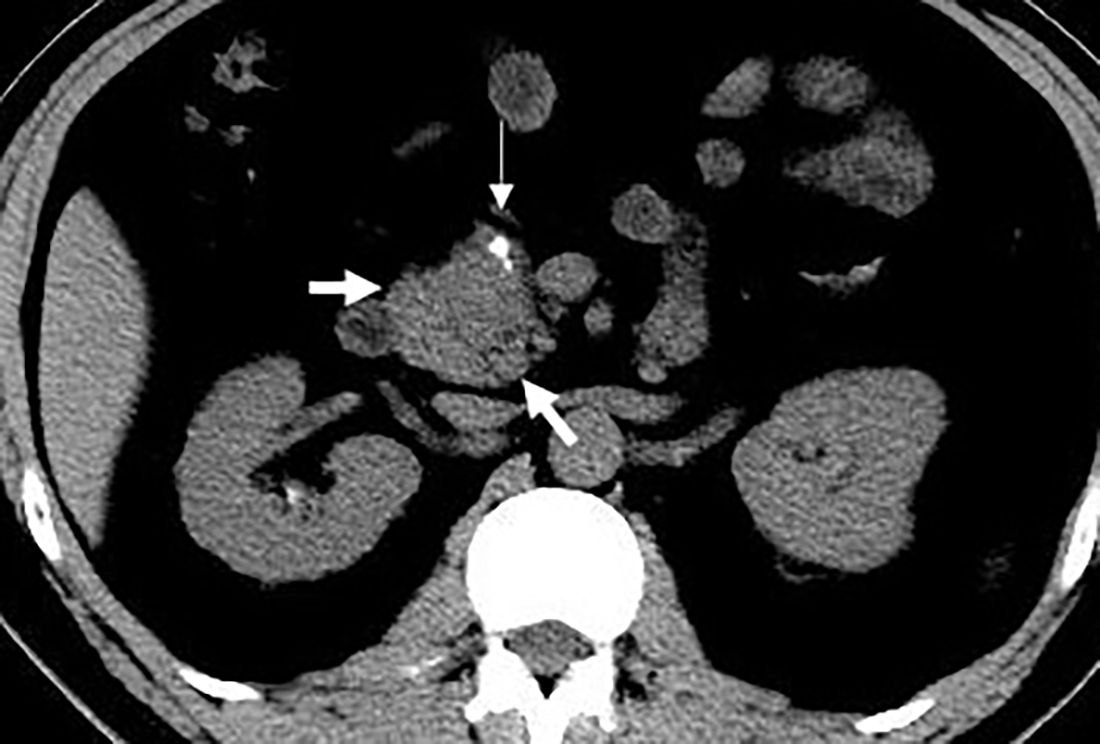

Supplement: Supplementary file 6 — Additional file 6. Supplementary Figure 6. PC in a patient with P-NEC. [file 40001_2022_725_MOESM6_ESM.zip › Supplementary figure 6-a.jpg]

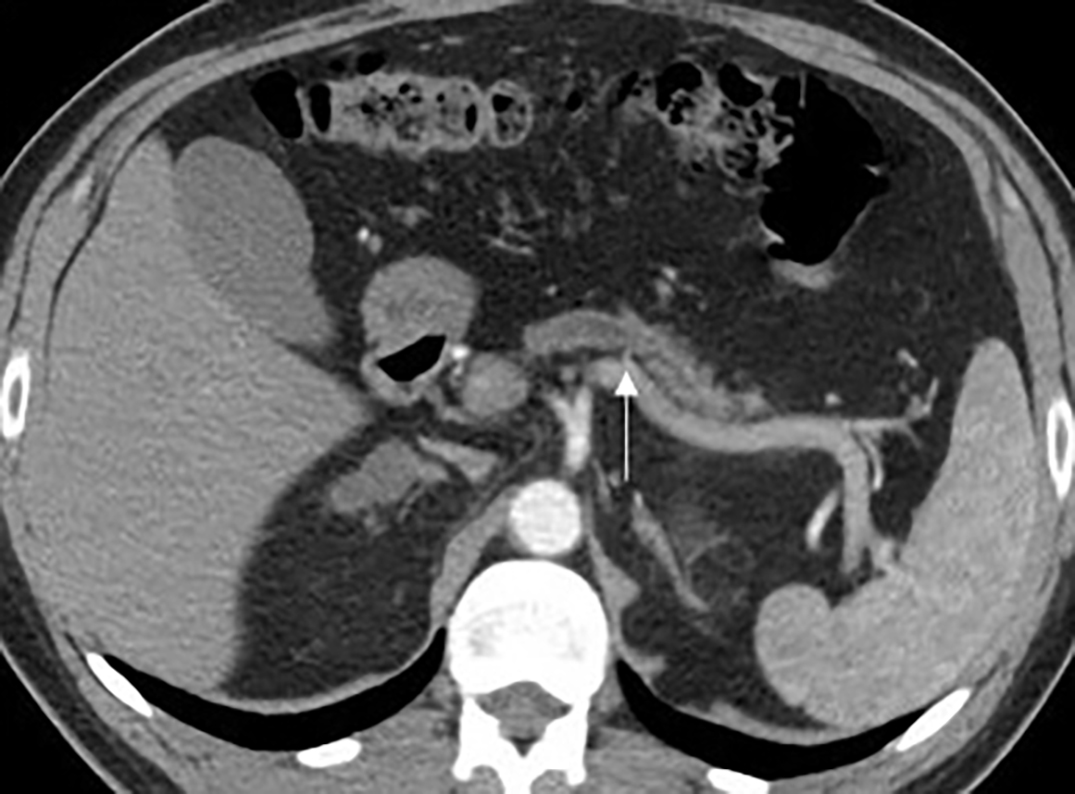

Supplement: Supplementary file 6 — Additional file 6. Supplementary Figure 6. PC in a patient with P-NEC. [file 40001_2022_725_MOESM6_ESM.zip › Supplementary figure 6-b.jpg]

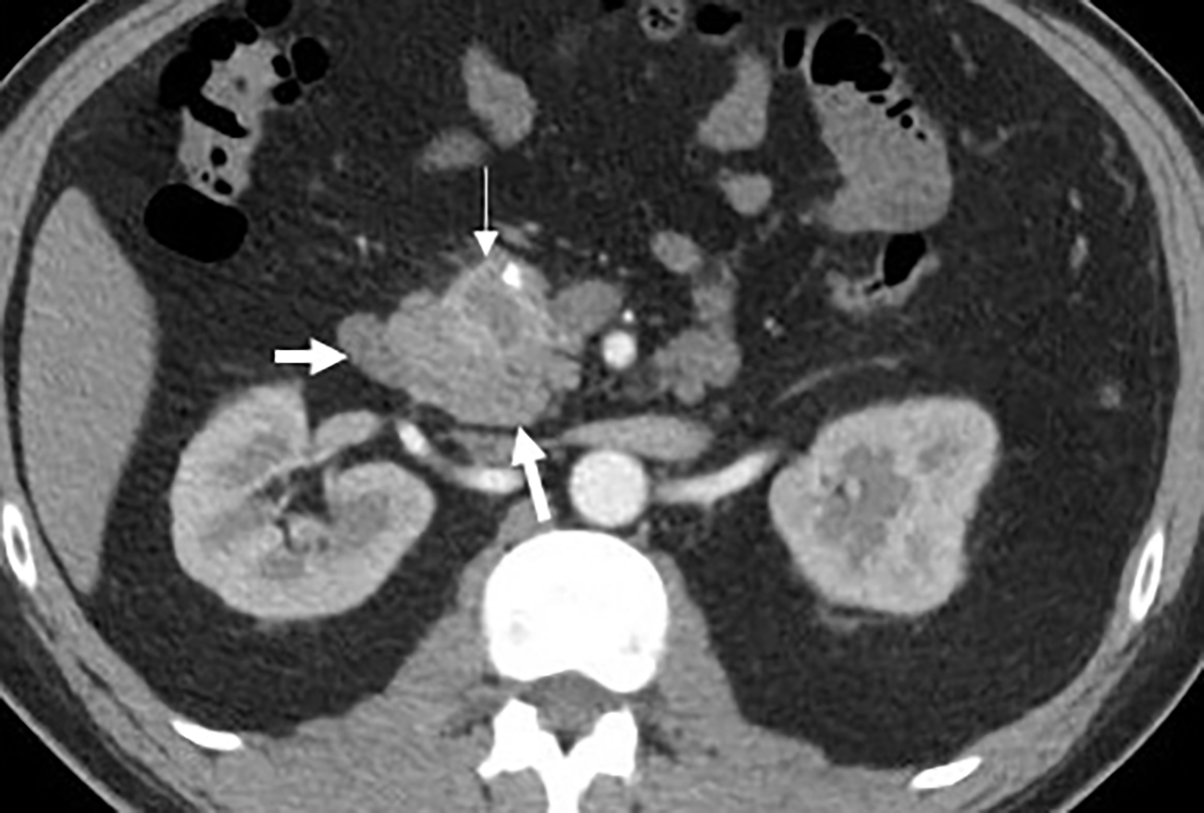

Supplement: Supplementary file 6 — Additional file 6. Supplementary Figure 6. PC in a patient with P-NEC. [file 40001_2022_725_MOESM6_ESM.zip › Supplementary figure 6-c.jpg]

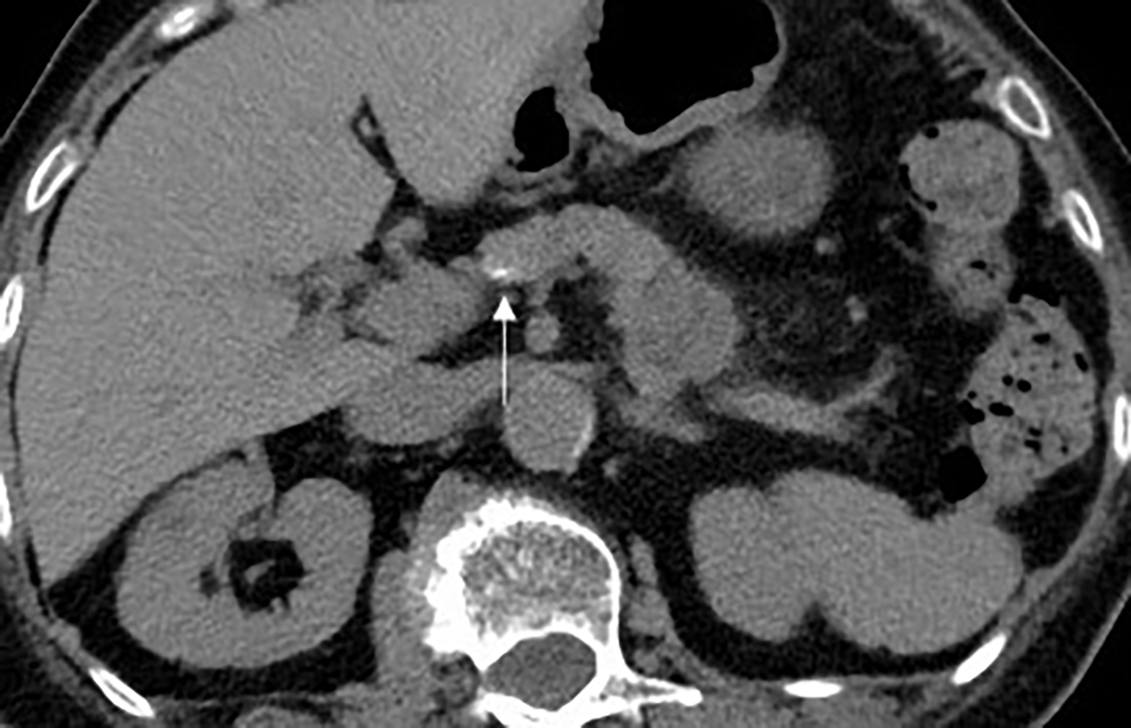

Supplement: Supplementary file 7 — Additional file 7. Supplementary Figure 7. PC in a patient with malignant IPMN. [file 40001_2022_725_MOESM7_ESM.zip › 1-Supplementary figure 7-a.jpg]

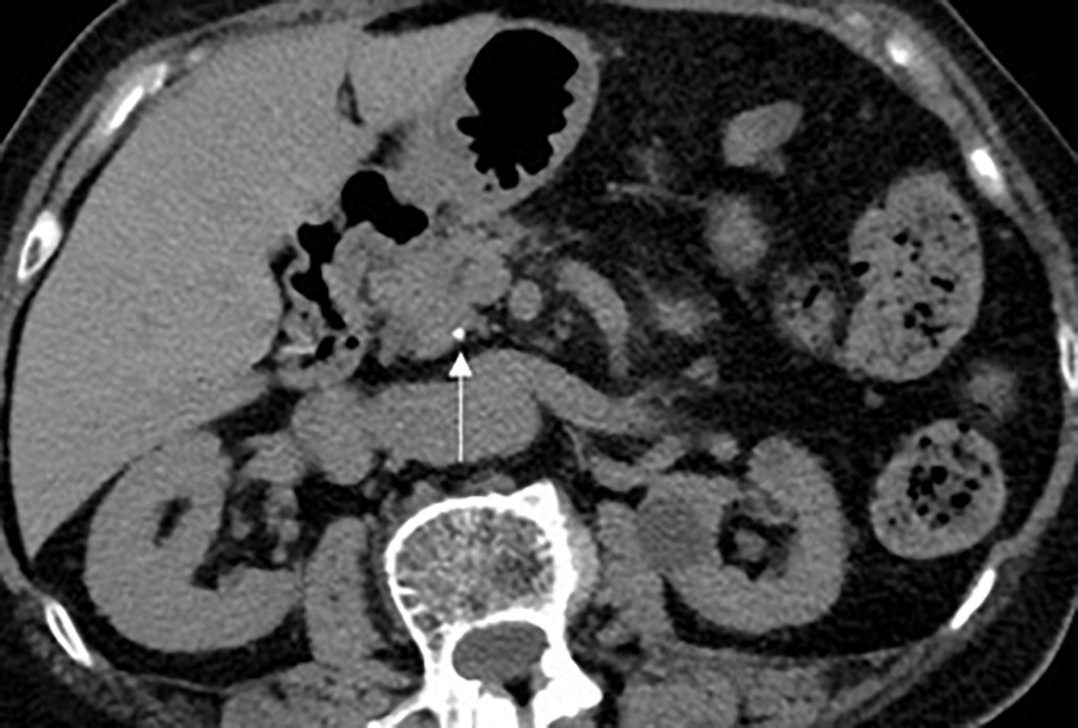

Supplement: Supplementary file 7 — Additional file 7. Supplementary Figure 7. PC in a patient with malignant IPMN. [file 40001_2022_725_MOESM7_ESM.zip › 1-Supplementary figure 7-b.jpg]

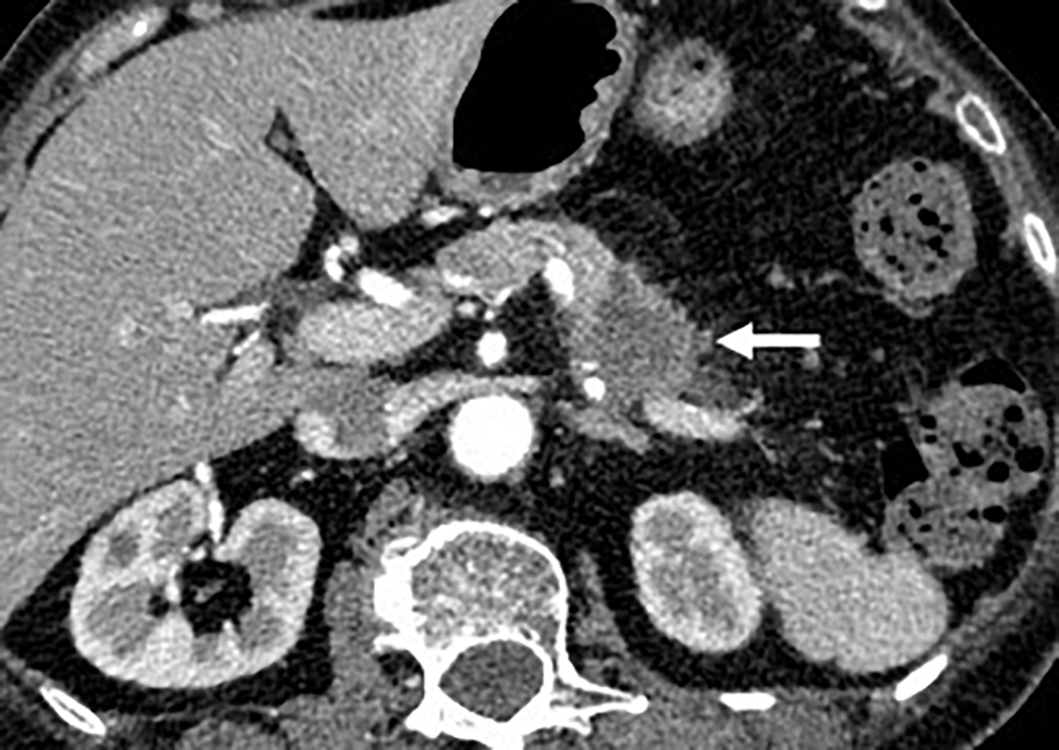

Supplement: Supplementary file 7 — Additional file 7. Supplementary Figure 7. PC in a patient with malignant IPMN. [file 40001_2022_725_MOESM7_ESM.zip › Supplementary figure 7-c.jpg]

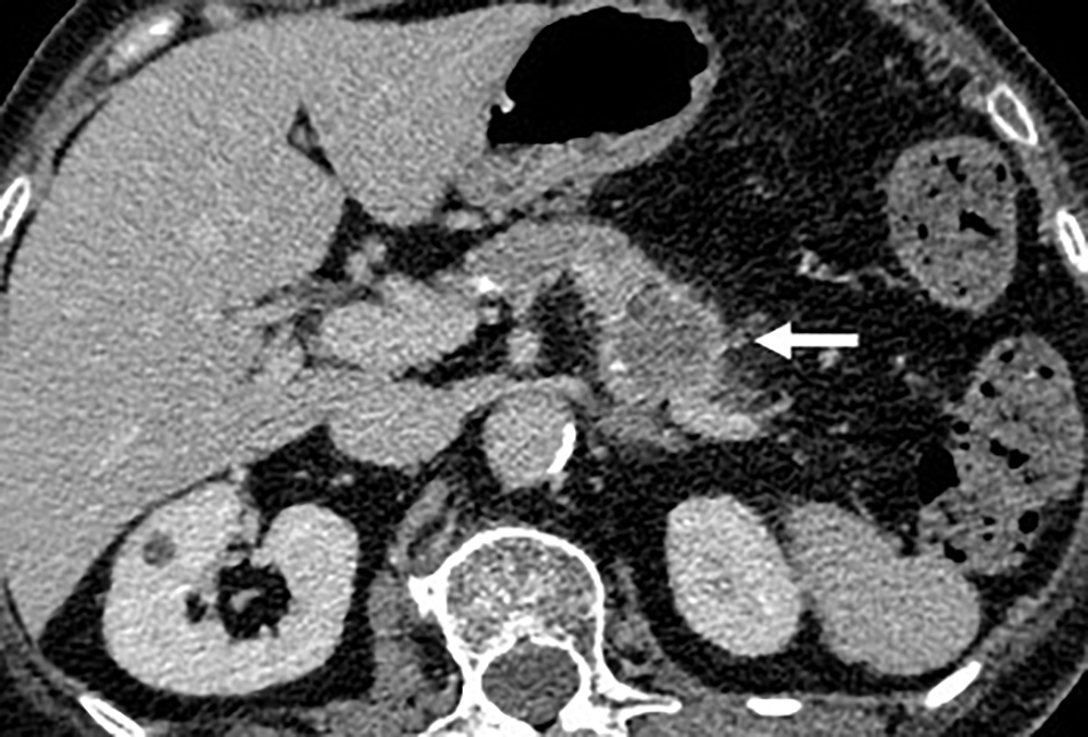

Supplement: Supplementary file 7 — Additional file 7. Supplementary Figure 7. PC in a patient with malignant IPMN. [file 40001_2022_725_MOESM7_ESM.zip › Supplementary figure 7-d.jpg]

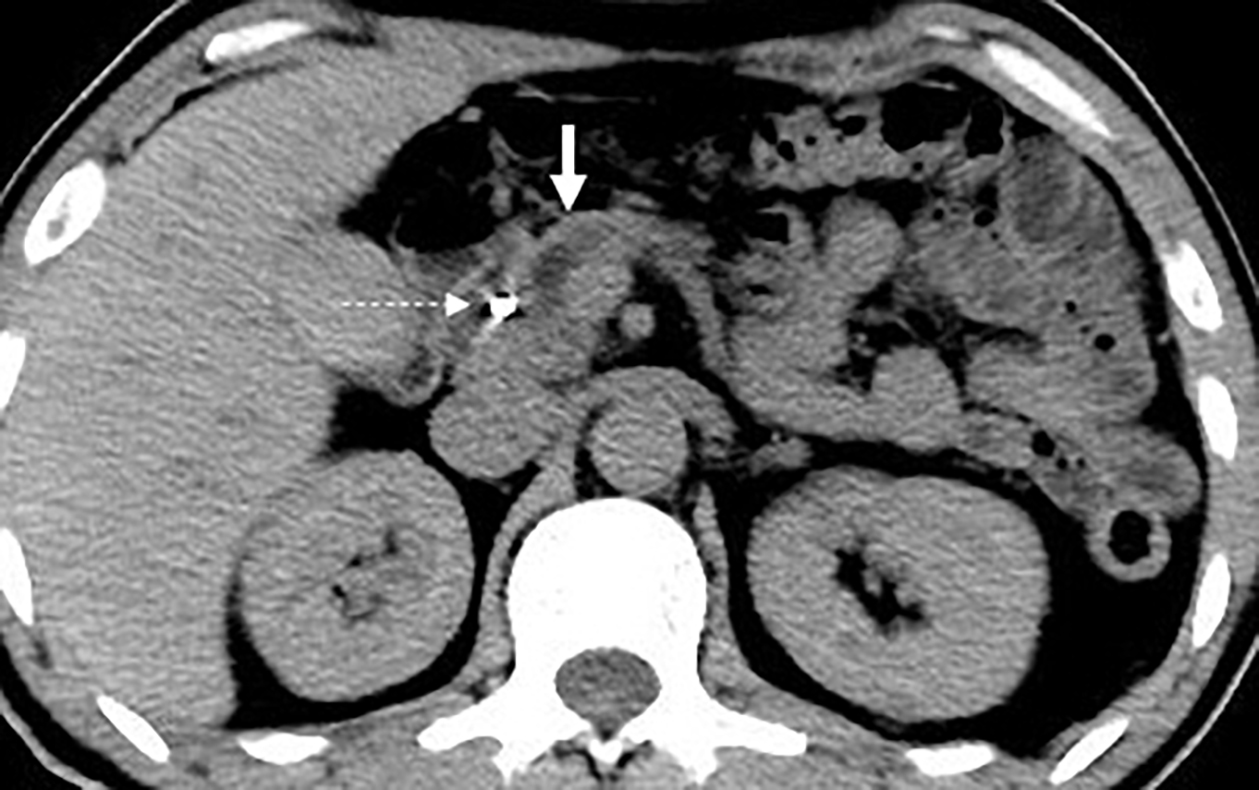

Supplement: Supplementary file 8 — Additional file 8. Supplementary Figure 8. PC in a patient with malignant IPMN. [file 40001_2022_725_MOESM8_ESM.zip › Supplementary figure 8-a.jpg]

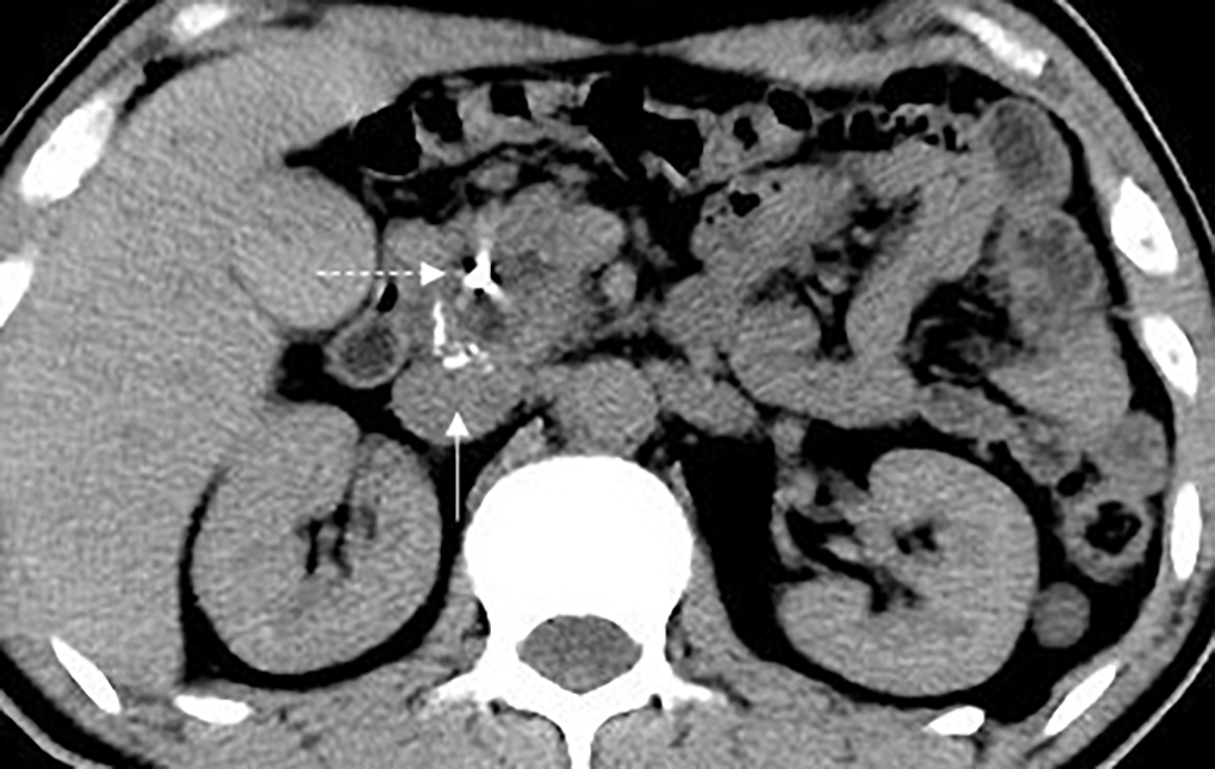

Supplement: Supplementary file 8 — Additional file 8. Supplementary Figure 8. PC in a patient with malignant IPMN. [file 40001_2022_725_MOESM8_ESM.zip › Supplementary figure 8-b.jpg]

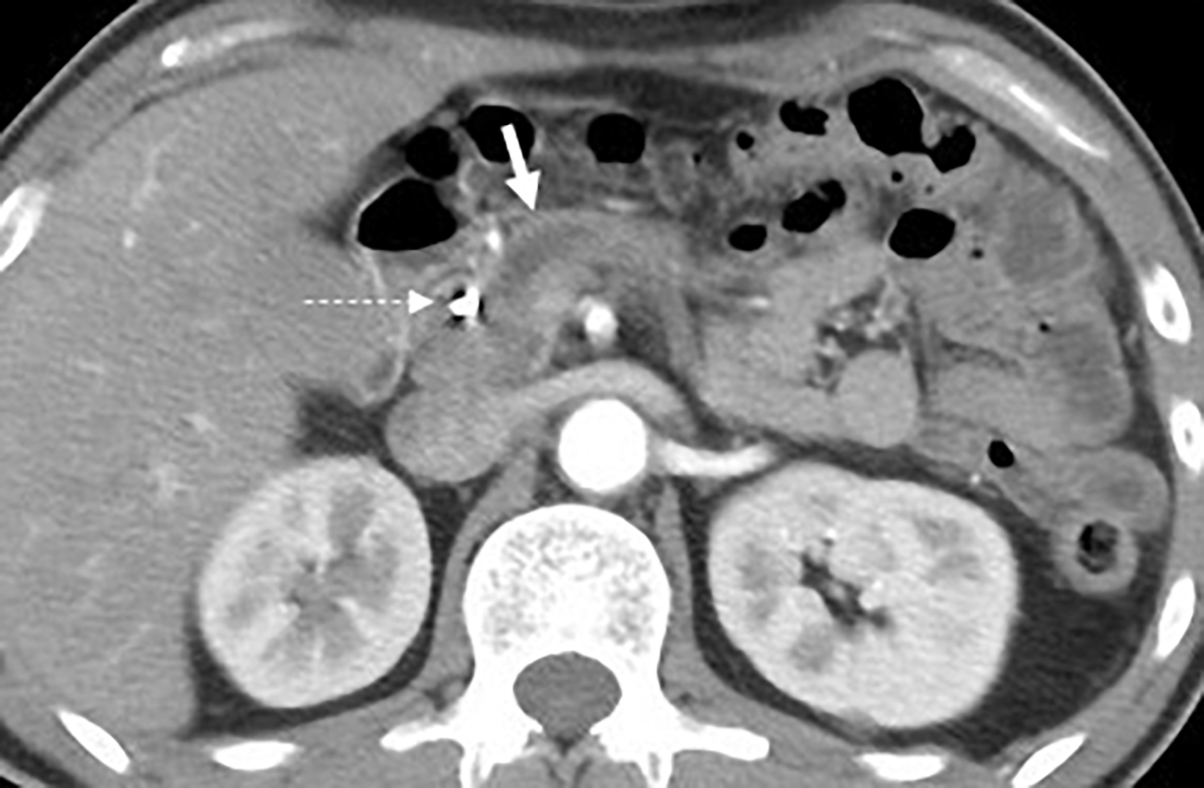

Supplement: Supplementary file 8 — Additional file 8. Supplementary Figure 8. PC in a patient with malignant IPMN. [file 40001_2022_725_MOESM8_ESM.zip › Supplementary figure 8-c.jpg]

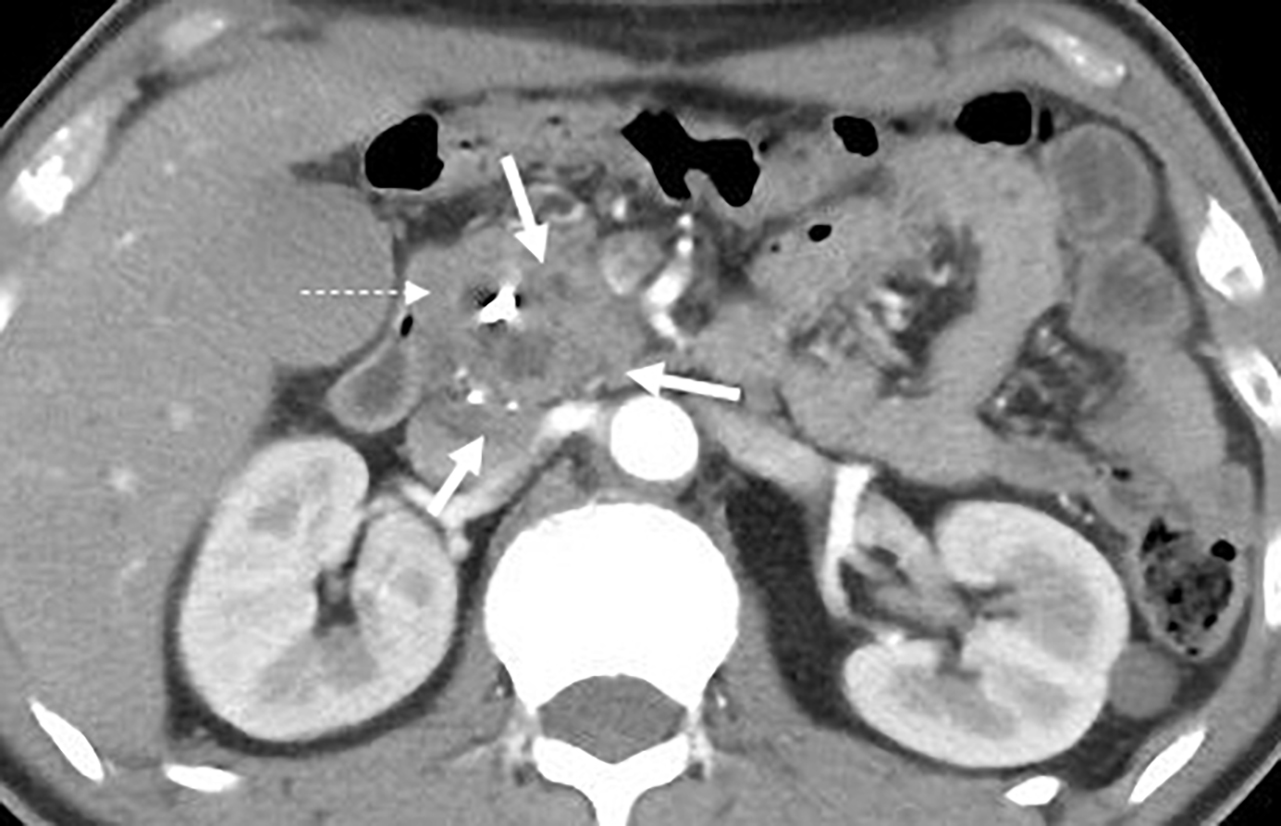

Supplement: Supplementary file 8 — Additional file 8. Supplementary Figure 8. PC in a patient with malignant IPMN. [file 40001_2022_725_MOESM8_ESM.zip › Supplementary figure 8-d.jpg]

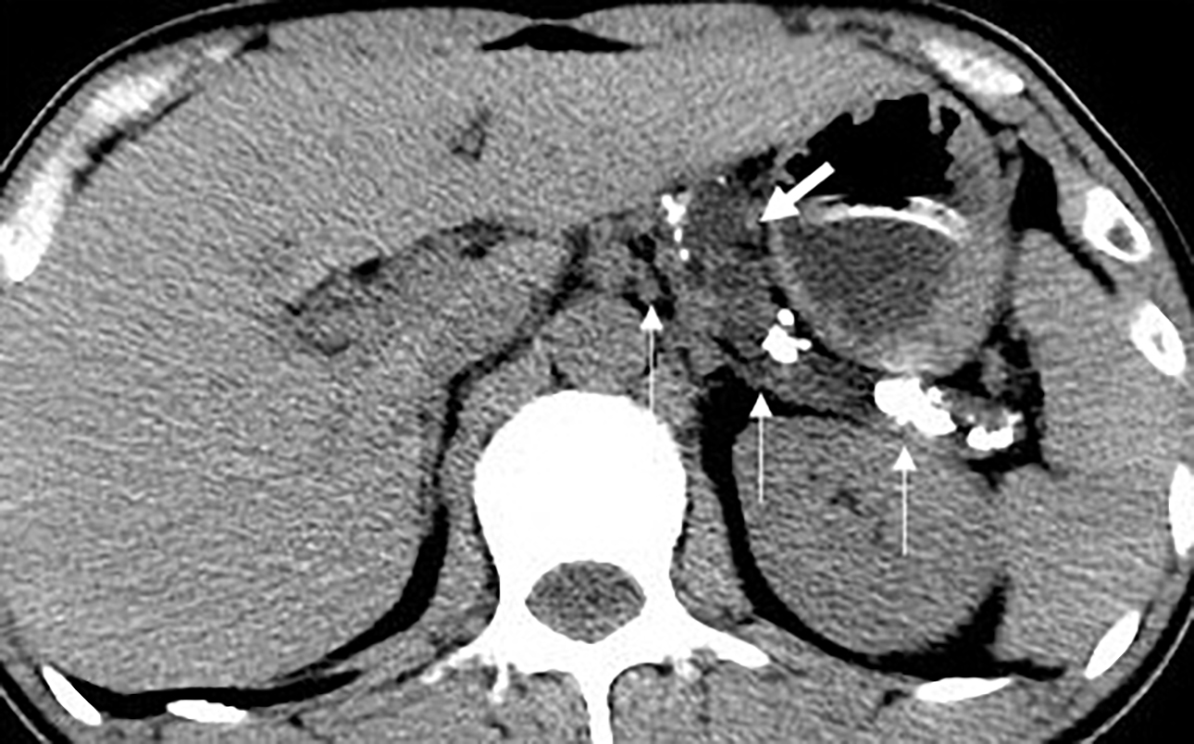

Supplement: Supplementary file 9 — Additional file 9. Supplementary Figure 9. PC in a patient with CP combined with PDAC. [file 40001_2022_725_MOESM9_ESM.zip › Supplementary figure 9-a.jpg]

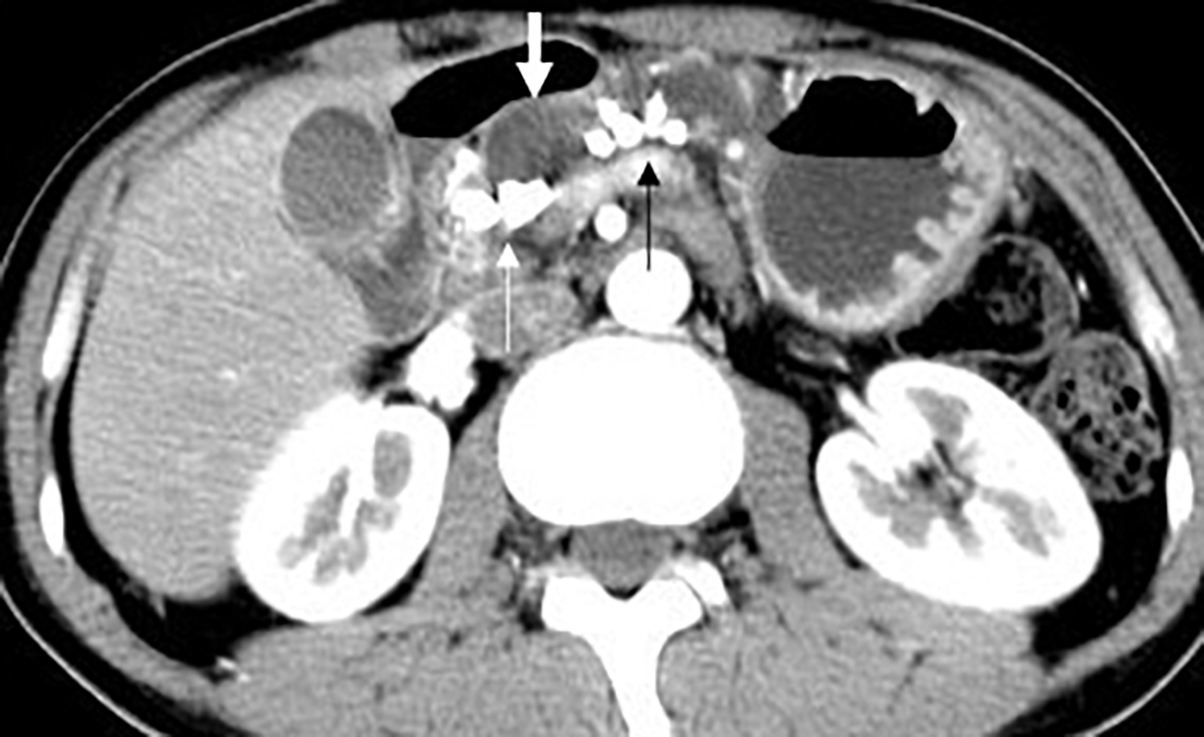

Supplement: Supplementary file 9 — Additional file 9. Supplementary Figure 9. PC in a patient with CP combined with PDAC. [file 40001_2022_725_MOESM9_ESM.zip › Supplementary figure 9-b.jpg]

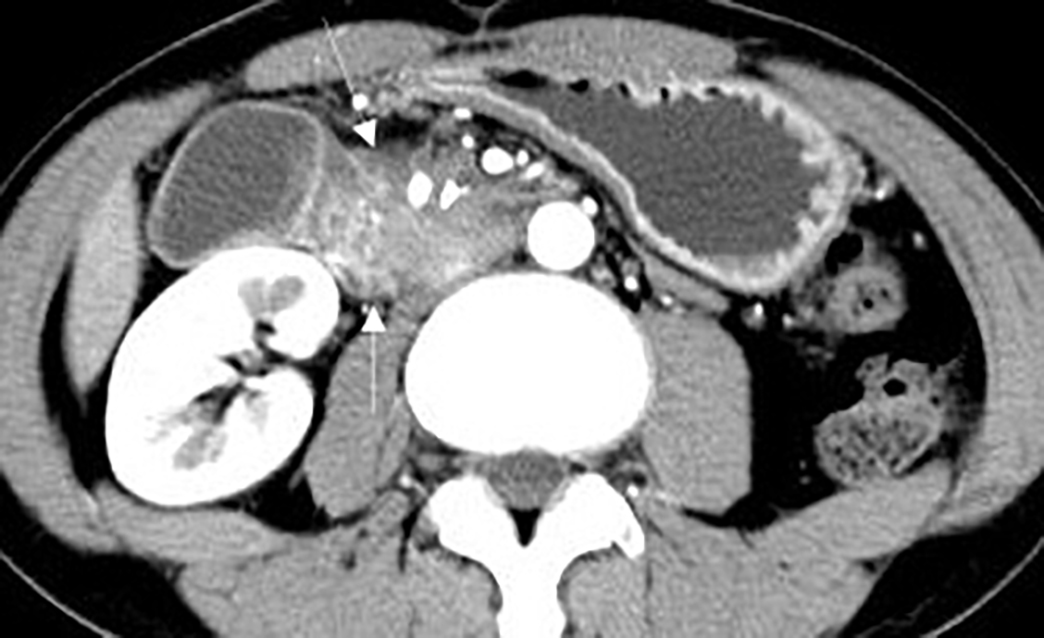

Supplement: Supplementary file 9 — Additional file 9. Supplementary Figure 9. PC in a patient with CP combined with PDAC. [file 40001_2022_725_MOESM9_ESM.zip › Supplementary figure 9-c.jpg]

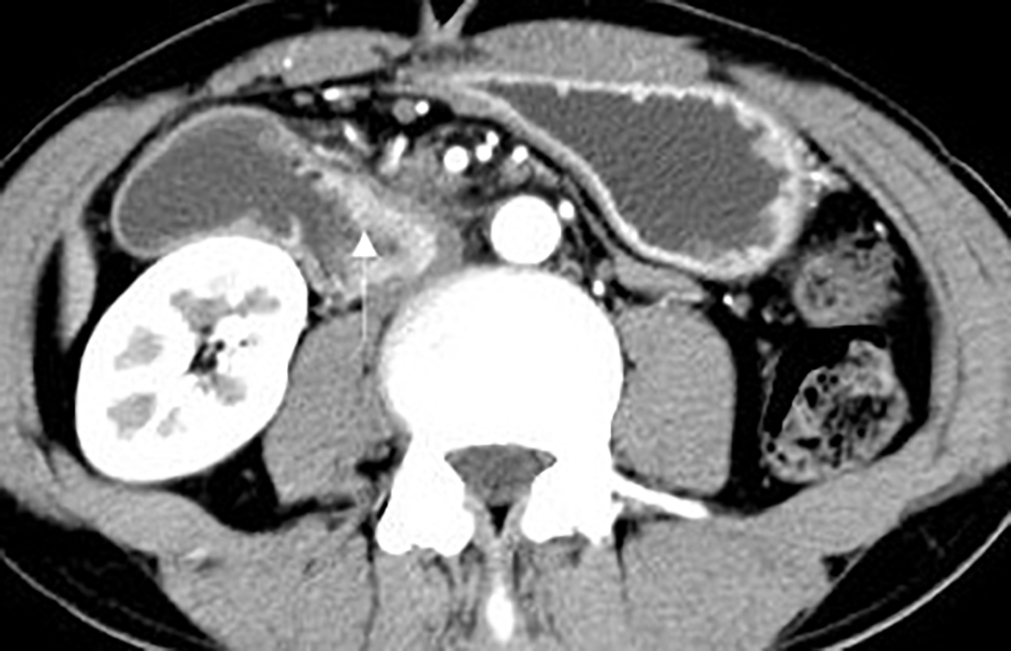

Supplement: Supplementary file 9 — Additional file 9. Supplementary Figure 9. PC in a patient with CP combined with PDAC. [file 40001_2022_725_MOESM9_ESM.zip › Supplementary figure 9-d.jpg]
