# Supplementary material for: Clinical significance of pancreatic calcifications: a 15-year single-center observational study
Source: Eur J Med Res. 2022 Jun 25;27:99. doi: 10.1186/s40001-022-00725-9 (PMC9233388; doi:10.1186/s40001-022-00725-9)
Supplement: Supplementary file 10 — Additional file 10. Supplementary Tables. [file 40001_2022_725_MOESM10_ESM.docx]

**Supplementary Tables**

| Table S1. The clinical characteristics of 204 patients with chronic pancreatitis | | | | | | |
| --- | --- | --- | --- | --- | --- | --- |
| Clinical characteristic | Non-malignant (n = 170) | |  | Malignant (n = 34) | | P Value |
|  | n | % |  | n | % |  |
| Age at admission (years) （mean±SD）^a^ | 51.4 ± 14.0 | |  | 61.6 ± 11.5 | | 0.0001 |
| Age at admission (years): >55 | 70 | 41.2 |  | 27 | 79.4 | 0.000 |
| Sex (male) | 120 | 70.6 |  | 25 | 73.5 | 0.730 |
| Body mass index (kg / m^2^ ) （mean±SD）^a^ | 21.8 ± 3.3 | |  | 22.2 ± 3.1 | | 0.6071 |
| Body mass index (kg / m^2^): >24 | 19 | 12.8 |  | 9 | 30.0 | 0.019 |
| Duration of symptoms before admission （mean±SD）^a^ | 17.5 ± 2.7 | |  | 3.9 ± 1.3 | | 0.0246 |
| Duration of symptoms before admission: >12 months | 44 | 25.8 |  | 2 | 5.9 | 0.012 |
| Risk factors |  |  |  |  |  |  |
| Smokers | 53 | 31.2 |  | 12 | 35.3 | 0.638 |
| Alcohol drinkers | 35 | 20.6 |  | 7 | 20.6 | 1.000 |
| Major symptoms |  |  |  |  |  |  |
| Chronic abdominal pain, back or lumbar pain | 119 | 70.0 |  | 21 | 61.8 | 0.345 |
| Steatorrhea or indigestion | 23 | 13.5 |  | 6 | 17.7 | 0.530 |
| Physical examination ^b^ | 23 | 13.5 |  | 6 | 17.7 | 0.530 |
| Black stool | 3 | 1.8 |  | 0 | 0.0 | 1.000 |
| Jaundice or high-colored urine | 15 | 8.8 |  | 7 | 20.6 | 0.043 |
| Case history |  |  |  |  |  |  |
| Diabetes mellitus | 38 | 22.4 |  | 16 | 47.1 | 0.003 |
| Gallstones or Cholecystectomy | 11 | 6.5 |  | 1 | 2.9 | 0.695 |
| Imaging |  |  |  |  |  |  |
| Pseudocysts | 36 | 21.2 |  | 3 | 8.8 | 0.149 |
| Pancreatic mass | 65 | 38.2 |  | 29 | 85.3 | 0.000 |
| Pancreatic mass located in the head of the pancreas | 45 | 26.5 |  | 19 | 55.9 | 0.001 |
| Pancreatic calcification | 98 | 57.7 |  | 25 | 73.5 | 0.084 |
| Pancreatic ductal dilatation^c^ | 110 | 70.1 |  | 22 | 64.7 | 0.540 |
| Atrophy of the parenchyma^c^ | 83 | 52.9 |  | 27 | 79.4 | 0.005 |
| CBD diameter (mean ± SD)^d^ | 8.8 ± 0.4 | |  | 10.1 ± 1.1 | | 0.2242 |
| CBD dilation or CBD stenting | 83 | 48.8 |  | 16 | 47.1 | 0.851 |
| Serological examination |  |  |  |  |  |  |
| CA 19-9 elevation^e^ | 38 | 24.4 |  | 20 | 58.8 | 0.010 |
| CA 125 elevation ^f^ | 17 | 11.3 |  | 7 | 21.2 | 0.124 |
| CEA elevation ^f^ | 24 | 16.0 |  | 12 | 35.3 | 0.010 |
| Total bilirubin elevation | 39 | 22.9 |  | 17 | 50.0 | 0.001 |
| ^a^: All but the three variables were variables for logistic regression; ^b^: Pancreatic mass, pancreatic calcification or serological CA19-9 elevation was found; ^c^: data of 13 patients were not available; ^d^: not included the 9 cases who had stent implanted in common bile duct (CBD) and 13 cases whose imagings were not performed in our hospital; ^e^: data of 14 patients were not available; ^f^: data of 20 patients were not available. | | | | | | |

| Table S2. Computed tomography findings in the study patients (CP vs. Non-CP) | | | | | | | | | |  |
| --- | --- | --- | --- | --- | --- | --- | --- | --- | --- | --- |
|  | Calcifications (%) | | | | Atrophy (%) | Pancreatic lesions (%) | | | Duct Dil. (%) | |
|  | Parenchymal only | | Intraductal | |  | Cystic | Solid | Cys+Sol |  |  |
| (n) | Diffuse. | Seg. | only | + Pare. |  |  |  |  |  | |
| CP (83) ^a^ | 4 (4.8) | 4 (4.8) | 6 (7.2) | 69^b^ (83.1) | 66 (79.5) | 18 (21.7) | 15 (18.1) | 3 (3.6) | 72 (86.7) | |
| CP+IPMN (2) | 0 | 0 | 1 (50) | 1 ^c^ (50) | 0 | 1 (50) | 0 | 0 | 1 (50) | |
| CP+PDAC (25) | 1 | 14 (56) | 0 | 10 ^c^ (40) | 21 (84) | 13 (52) | 0 | 8 (32) | 16 (64) | |
| SPT (15) | 0 | 15 (100) | 0 | 0 | 0 | 2 (13.3) | 4 (26.7) | 6 (40) | 0 | |
| SCN (12) | 0 | 12 (100) | 0 | 0 | 2 (16.7) | 12 (100) | 0 | 0 | 1 (8.3) | |
| P-NN (10) | 2 (20) | 8 ^d^ (80) | 0 | 0 | 1 (10) | 0 | 9 ^d^(90) | 1(10) | 1 ^d^ (10) | |
| IPMN（5 ^e^） | 1 (20) | 3 (60) | 1 (20) | 0 | 5 (100) | 1 (20) | 1 (20) | 2 (40) | 3 (60) | |
| MCN (3) | 0 | 3 (100) | 0 | 0 | 1 ^e^(33.3) | 3 (100) | 0 | 0 | 1 ^e^ (33.3) | |
| ^a^: not included 13 patients whose CT imagings before operation were performed in other hospital. ^b^: Diffuse distribution was found in 62 cases. ^c^: Diffuse distribution was found in all cases. ^d^: included one patient with mixed ductal-endocrine carcinoma of the pancreas. ^e^: Malignant. Abbreviations: Cys+Sol, the pancreatic lesion has the features of Cystic and the one of Solid; Seg., Segmental; Pare., Parenchymal. Dil., Dilatation. Abbreviations: CP, chronic pancreatitis; IPMN, Intraductal Papillary Mucinous Neoplasm; PDAC, Pancreatic ductal adenocarcinoma; SPT, Solid pseudopapillary tumors; SCN, Serous Cystic Neoplasm; P-NN: Pancreatic neuroendocrine neoplasm; MCN, Mucinous Cystic Neoplasm. | | | | | | | | | |  |

| Table S3. Sensitivity and specificity of pancreatic calcifications (n = 155 ^a^) in the diagnosis of CP and the diagnosis of CP or IPMN. | | |  |
| --- | --- | --- | --- |
|  | Sensitivity | Specificity |  |
| The diagnosis of CP ^b^ |  |  |  |
| Parenchymal calcifications only | 20.9% (23/110) | 2.22% (1/45) |  |
| Diffuse distribution | 4.55% (5/110) | 93.3% (42/45) |  |
| Segmental distribution | 16.4% (18/110) | 8.89% (4/45) |  |
| Intraductal stones | 79.1% (87/110) | 97.8% (44/45) |  |
| Intraductal stones only | 6.36% (7/110) | 97.8% (44/45) |  |
| Both intraductal & parenchymal calcifications | 72.7% (80/110) | 100% (45/45) |  |
| The diagnosis of Malignant diseases ^c^ |  |  | |
| Parenchymal calcifications only | 19.1% (21/110) | 62.6% (77/123) | |
| Diffuse distribution | 6.3% (2/32) | 95.1% (117/123) | |
| Segmental distribution | 59.4% (19/32) | 67.5% (83/123) | |
| Intraductal stones | 34.3% (11/32) | 37.4% (46/123) | |
| Intraductal stones only | 3.1% (1/32) | 94.3% (116/123) | |
| Combined with Parenchymal calcifications | 31.3% (10/32) | 43.1% (53/123) | |
| ^a^: not included 13 patients whose imaging was performed in other hospital. ^b^: included simple CP (n = 83), CP combined with IPMN (n = 2) and CP combined with PDAC (n = 25). ^c^: included CP combined with PDAC (n = 25), malignant IPMN (n = 5), mixed ductal-endocrine carcinoma of the pancreas (n = 1) and mucinous cystadenocarcinoma (n = 1). | | | |

| Table S4. The imaging findings of 81^a^ patients with pancreatic mass | | | | | | |
| --- | --- | --- | --- | --- | --- | --- |
| Clinical characteristic | Non-malignant  (n = 53) | |  | Malignant  (n = 28) | | *p* Value |
|  | n | % |  | n | % |  |
| Maximum diameter (mean ± SD) ^b^ | 3.2 ± 1.7 | |  | 3.7 ± 1.8 | | 0.2291 |
| Maximum diameter ≥3.5cm | 22 | 41.5 |  | 17 | 60.7 | 0.100 |
| Pancreatic calcification | 12 | 22.6 |  | 21 | 75.0 | 0.000 |
| Pancreatic ductal dilatation | 33 | 62.3 |  | 17 | 60.7 | 0.891 |
| Atrophy of the parenchyma | 18 | 34.0 |  | 22 | 78.6 | 0.000 |
| Common bile duct (CBD) dilation or CBD stent implantation | 34 | 64.2 |  | 13 | 46.4 | 0.124 |
| CBD diameter (mean ± SD) ^b^ | 9.3 ± 4.9 | |  | 10.2 ± 7.1 | | 0.5239 |
| Location：head | 37 | 69.8 |  | 18 | 64.3 | 0.612 |
| Suspicious lymph nodes ^c^ | 11 | 20.8 |  | 4 | 14.3 | 0.560 |
| Abutting or encasing the arteries around the pancreas | 12 | 22.6 |  | 12 | 42.9 | 0.058 |
| Abutting or invading the veins around the pancreas | 18 | 34.0 |  | 18 | 64.3 | 0.009 |
| ^a^: not included the 5 cases with pancreatic pseudocyst, 6 cases whose imaging were performed in other hospital, and 2 cases combined with IPMN. ^b^: All but the two variables are regression variables. ^c^: In the non-malignant group, 11 cases were found to have suspicious lymph nodes, located at the porta hepatis (n = 8), the porta hepatis and around the pancreas (n = 2), and the area of splenic hilar region (n = 1). In the malignant group, 4 cases were found to have suspicious lymph nodes, located at the porta hepatis (n = 2), the paraaortic area (n = 1), and at multiple areas including porta hepatis, celiac trunk, peripancreatic, paraaortic, pericaval, and mesenteric area (n = 1). | | | | | | |
